# Inflammation, Anti-inflammatory Interventions, and Post-stroke Cognitive Impairment: a Systematic Review and Meta-analysis of Human and Animal Studies

**DOI:** 10.1007/s12975-023-01218-5

**Published:** 2023-11-28

**Authors:** Reinier W. P. Tack, Claudia Amboni, Danny van Nuijs, Marcela Pekna, Mervyn D. I. Vergouwen, Gabriel J. E. Rinkel, Elly M. Hol

**Affiliations:** 1 Department of Neurology and Neurosurgery, UMC Utrecht Brain Centre, University Medical Centre Utrecht, Utrecht University, Heidelberglaan 100, 3584 CX Utrecht, The Netherlands; 2 Department of Translational Neuroscience, UMC Utrecht Brain Centre, University Medical Centre Utrecht, Utrecht University, Heidelberglaan 100, 3584 CX Utrecht, The Netherlands; 3 Laboratory of Regenerative Neuroimmunology, Center for Brain Repair, Department of Clinical Neuroscience, Institute of Neuroscience and Physiology, Sahlgrenska Academy at the University of Gothenburg, Gothenburg, Sweden

**Keywords:** Stroke, Inflammation, Cognitive impairment, Microglia, Complement

## Abstract

The pathophysiology and treatment of post-stroke cognitive impairment (PSCI) are not clear. Stroke triggers an inflammatory response, which might affect synapse function and cognitive status. We performed a systematic review and meta-analysis to assess whether patients with PSCI have increased levels of inflammatory markers and whether anti-inflammatory interventions in animals decrease PSCI. We systematically searched PubMed, EMBASE, and PsychInfo for studies on stroke. For human studies, we determined the standardized mean difference (SMD) on the association between PSCI and markers of inflammation. For animal studies, we determined the SMD of post-stroke cognitive outcome after an anti-inflammatory intervention. Interventions were grouped based on proposed mechanism of action. In patients, the SMD of inflammatory markers for those with versus those without PSCI was 0.46 (95% CI 0.18; 0.76; *I*^2^ = 92%), and the correlation coefficient between level of inflammation and cognitive scores was − 0.25 (95% CI − 0.34; − 0.16; *I*^2^ = 75%). In animals, the SMD of cognition for those treated with versus those without anti-inflammatory interventions was 1.43 (95% CI 1.12; 1.74; *I*^2^ = 83%). The largest effect sizes in treated animals were for complement inhibition (SMD = 1.94 (95% CI 1.50; 2.37), *I*^2^ = 51%) and fingolimod (SMD = 2.1 (95% CI 0.75; 3.47), *I*^2^ = 81%). Inflammation is increased in stroke survivors with cognitive impairment and is negatively correlated with cognitive functioning. Anti-inflammatory interventions seem to improve cognitive functioning in animals. Complement inhibition and fingolimod are promising therapies on reducing PSCI.

**Supplementary Information:**

The online version contains supplementary material available at 10.1007/s12975-023-01218-5.

## Introduction

Fifty percent of stroke survivors have cognitive impairment [1]. While several risk factors for the development of post-stroke cognitive impairment (PSCI) have been identified, the underlying mechanisms remain unclear [2]. Inflammation following stroke has been proposed as a contributor to the pathogenesis of PSCI [3].

After both ischemic and hemorrhagic stroke, a vast array of cellular (infiltration of immune cells, activation of microglia) and molecular (increased expression of chemokines or interleukins) inflammatory changes occur [4–6]. Under the influence of inflammatory changes, reactive microglia, and subsequently reactive astrocytes can impact synapse function by increased synaptic pruning or decreased synaptic plasticity, which in turn may lead to cognitive impairment [7]. Clinical studies on the relationship between inflammation and cognition after stroke show conflicting results, which can be explained by small sample sizes and different outcome measures [8–11]. Although some animal studies using anti-inflammatory drugs showed promising results in preventing PSCI, it remains unclear whether in animals a relation exists between inflammatory changes and PSCI, and if so, which inflammatory cells or mediators are involved in PSCI pathogenesis [12]. Likewise, it remains unclear whether anti-inflammatory interventions decrease PSCI, and if so which interventions do so. Therefore, we performed a translational systematic review and meta-analysis to determine whether stroke survivors with PSCI have higher levels of inflammatory markers than those without PSCI, and whether anti-inflammatory interventions in animals decrease PSCI.

## Methods

### Design, Registration, and Reporting

This systematic review was designed following the Joanna Briggs Institute guidelines on association studies [13]. An exploratory search of the literature showed that human studies are mainly observational linking inflammatory read-outs to cognitive scores and animal studies are focused on testing the effect of specific anti-inflammatory interventions on brain damage and behavior. To address this and to allow for a single search strategy, we used inflammation as a dependent variable in analyzing human study results and as an independent variable in analyzing animal study results. We used human studies to assess whether inflammatory markers are higher in stroke patients with cognitive impairment than in those without, and animal studies to assess whether anti-inflammatory interventions decrease cognitive impairments after stroke. Human and animal data systematic review designs were registered separately in PROSPERO (identifiers CRD42021176786 and CRD42021210875) [14] using the preferred reporting items for systematic reviews and meta-analyses for protocols 2015 (PRISMA-P) [15].

### Search Strategy

This review was conducted in accordance with the preferred reporting items for systematic reviews and meta-analyses (PRISMA) statement [16]. We systematically searched the PubMed, Embase, and PsychInfo databases using broad, predefined synonyms for stroke, cognition, and inflammation. The search syntax can be found in “Supplementary materials”. The search was performed on April 3, 2020, and updated on April 5, 2021, and on August 31, 2023. A broad definition of inflammation was initially included in the search strategy and, after discussion with the co-authors, subsequently narrowed down to include only markers or interventions with an effect through a known inflammatory pathway. Any uncertainties were resolved in consensus meetings between the authors.

### In- and Exclusion Criteria

For human studies we used the following inclusion criteria: (1) cohort or case–control studies written in English; (2) studies including patients with ischemic and/or hemorrhagic stroke; (3) studies that compared concentrations of inflammatory markers in serum or cerebrospinal fluid (CSF) between patients with and without PSCI, or correlations between cognitive scores and concentrations of inflammatory markers; and (4) studies that evaluated cognition with a known cognitive screening tool or neuropsychological evaluation, regardless of cut-off value. If studies used vascular dementia (VaD) as an outcome measure for post-stroke cognitive impairment, the study was only included if the stroke had occurred before the onset of dementia. If PSCI and VaD were included as separate groups, outcomes were combined into a single group. Exclusion criteria were (1) studies including patients with a transient ischemic attack, (2) studies reporting on cognitive decline over time rather than cognitive impairment at a single time point, and (3) studies where > 10% of patients had cognitive impairment prior to stroke.

For animal studies the inclusion criteria were (1) studies written in English; (2) studies using a previously validated model of focal ischemic or hemorrhagic stroke; (3) studies investigating the effect of interventions (either administration of drugs or use of genetically modified animals) on inflammation through a known inflammatory pathway; (4) studies that used one of the following tests to assess cognitive functioning: Morris water maze, Barnes maze, Y-maze, T-maze, radial arm water maze, fear conditioning, avoidance test, novel object recognition, and object location task; and (5) studies that made a direct comparison of cognitive outcomes between stroke animals with and without pro/anti-inflammatory interventions. We excluded outcomes where cognitive deficits could be attributed to motor function impairment, such as swim speed differences in Morris water maze. We excluded all global ischemia models of ischemic stroke, such as 2-vessel occlusion and 4-vessel occlusion models, as we feel these are not representative of our population of interest.

### Qualitative Assessment

For human studies, we used a modified version of the Newcastle–Ottawa scale (NOS) for both quality assessment and risk of bias assessment [17]. For animal studies, we used SYRCLE’s risk of bias tool [18, 19]. The qualitative assessment was performed by two individual reviewers independently from each other (CA, DN). Quality assessment beyond 2021 was performed by DN and RWPT. Any discrepancies were resolved in consultation with the neurologists GJER and MDIV for the human studies and the neuroscientists MP and EMH for the animal studies.

### Data Extraction

For the human studies, we extracted the following variables: author and year of publication, type of stroke, inflammatory parameter, cognitive test and cut-off point, days between stroke and measure of inflammation, and days between stroke and cognitive testing.

For the animal studies, we extracted the following variables: author and year of publication, stroke model, animal species and strain, age and sex, inflammatory intervention, cognitive test, time between stroke and intervention or intervention and stroke (in case of intervention prior to stroke induction), time between stroke and cognitive testing, sample size, and outcome. If different dosages of an intervention were described, only the dosage with the largest effect size was included. Interventions administered at different time points were included as separate interventions. If a study included multiple biomarker measurements at different timepoints, we only included the initial measurement. To evaluate learning and retention in learning tasks, we only included data from the 3rd day after stroke (learning) and the final day of testing (retention).

Outcomes were extracted from the text if possible. If raw outcomes were not available or insufficient from the text or supplementary materials, the outcomes were extracted from the figures using WebPlotDigitizer version 4.5 (https://automeris.io/WebPlotDigitizer/). Using this tool, outcomes were extracted by two independent reviewers (RWPT and CA) and averaged before analysis. Beyond 2021, outcomes were extracted by a single reviewer (RWPT).

### Data Synthesis

Data synthesis was performed using R version 4.0.4 and package “esc”. For human studies, we determined the standardized mean difference (SMD) of post-stroke cognitive impairment on the level of inflammation measured. For animal studies, we determined the effect size of either a pro- or anti-inflammatory intervention on post-stroke cognitive outcome. The SMD (Hedges’ g) was used as effect size to control for potential bias due to the small study size and is reported in conjunction with the upper/lower limit of the 95% confidence interval (CI). In both the human and animal studies, SMD was considered small at 0.2, medium at 0.5, and large at 0.8 [20]. Effect sizes and standard errors were first calculated from the raw outcome variables. Outcomes were initially converted into effect sizes as described earlier [21]. If only median and interquartile range (IQR) or overall range were reported, mean and standard deviation (SD) were estimated as previously described before calculating effect size [22]. The direction of the effect size was reversed in animal cognitive tests where a higher score represented worse cognitive functioning, such as escape latency in Morris water maze. The direction of the effect size was reversed in pro-inflammatory models.

### Meta-analysis

The meta-analysis was performed using the R packages “dmetar”, “devtools”, and “meta” [21]. Given the expected heterogeneity, a random-effects model was chosen for all comparisons. Heterogeneity was calculated using Cochran’s *Q*-test and *I*^2^. We considered *I*^2^ to represent a low heterogeneity at 25%, moderate at 50%, and high at 75% [23]. Publication bias was assessed by visual examination of funnel plots and by Egger’s test, which we considered statistically significant if *p* < 0.1.

Primary analyses were performed by pooling study results to assess the association between PSCI and markers of inflammation. A three-level meta-analysis was performed to evaluate the distribution of variance across levels. If > 25% of heterogeneity was attributable to the within-study heterogeneity, we compared the three-level meta-analysis to a two-level analysis using analysis of variance (ANOVA). If the three-level model showed a better fit (*p* < 0.05), this model was used for the primary analysis. In the three-level meta-analysis, the R package “MAd” was used to aggregate different within-study outcomes, which implements previously described methods for data aggregation [24, 25]. We performed a meta-regression to identify the role of potential confounders (mean timing of cognitive assessment, mean timing of intervention, mean timing of inflammatory measure, cognitive test used, animal species). Bonferroni-correction was used to adjust for multiple testing. Subgroup analyses of independent inflammation markers or interventions were performed if these were represented in at least three independent studies. In animal studies, interventions were grouped based on the inflammatory pathway. All meta-regression and subgroup analyses were performed using two-level meta-analysis to prevent loss of information.

## Results

**Fig. 1:**
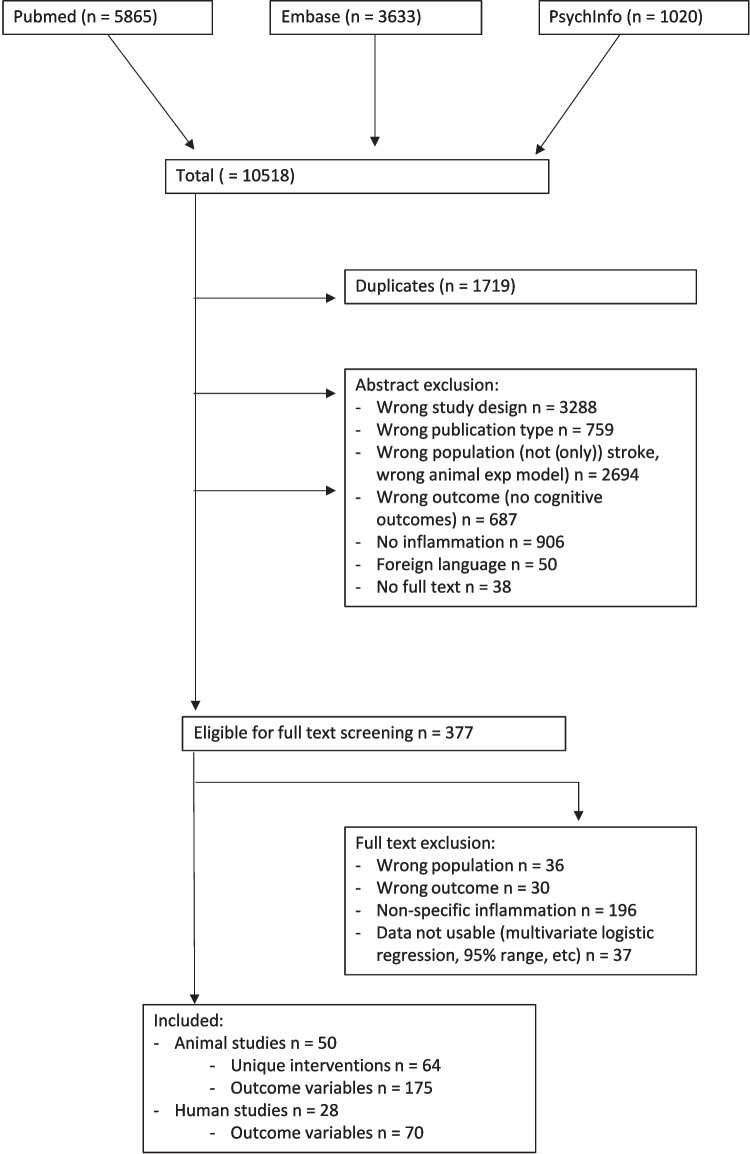
Flowchart of included studies

After title and abstract screening of 10,518 studies, 377 studies were selected for full-text examination, of which 78 were included (Fig. 1). Results of the search strategy, labeling, and reasons for exclusion are openly accessible (https://rayyan.ai/reviews/127225, https://rayyan.ai/reviews/264074 and https://rayyan.ai/reviews/765172). Of the 78 included studies, 28 were on ischemic stroke patients with a total of 70 outcome variables, and 50 were animal studies and included 64 unique interventions and 175 outcome variables.

### Characteristics of Included Studies

Characteristics of included studies are shown in Supplementary Tables 1–3. All human studies evaluated the association between inflammation and cognition in ischemic stroke. Of these studies, 13 used cut-offs of the mini-mental state examination (MMSE) or Montreal cognitive assessment (MoCA) tests to define PSCI, while the remaining 4 used a neuropsychological examination, six-item screener, or a VaD assessment scale. In one human study inflammatory markers (IL-1b and IL-10) were measured in CSF [26], in all other human studies inflammatory markers were measured in serum. Animal studies evaluated the effect of interventions targeting inflammation on cognition in ischemic stroke (*n* = 34), subarachnoid hemorrhage (*n* = 7), or intracerebral hemorrhage (*n* = 9). Interventions were diverse, targeting multiple aspects of the inflammatory cascade. Cognitive functioning was evaluated through Morris water maze (*n* = 112), Barnes maze (*n* = 14), radial arm water maze (*n* = 13), novel object recognition (*n* = 8), contextual fear memory (*n* = 3), cued fear memory (*n* = 1), T-maze (*n* = 1), Y-maze (*n* = 4), passive avoidance test (*n* = 1), and active avoidance test (*n* = 1).

### Quality Assessment

Supplementary Tables 4 and 5 show the risk of bias and quality assessment of the included studies. In human studies, the risk of bias was low (score 7–9) in 11 studies and high (score 4–6) in 17 studies. In the included animal studies, the overall risk of bias was low to moderate. Potential sources of bias were the baseline characteristics, housing, random outcome assessment, reporting of drop-outs, and selective outcome reporting.

### Inflammatory Parameters in Stroke Patients with and Without Cognitive Impairment

**Fig. 2:**
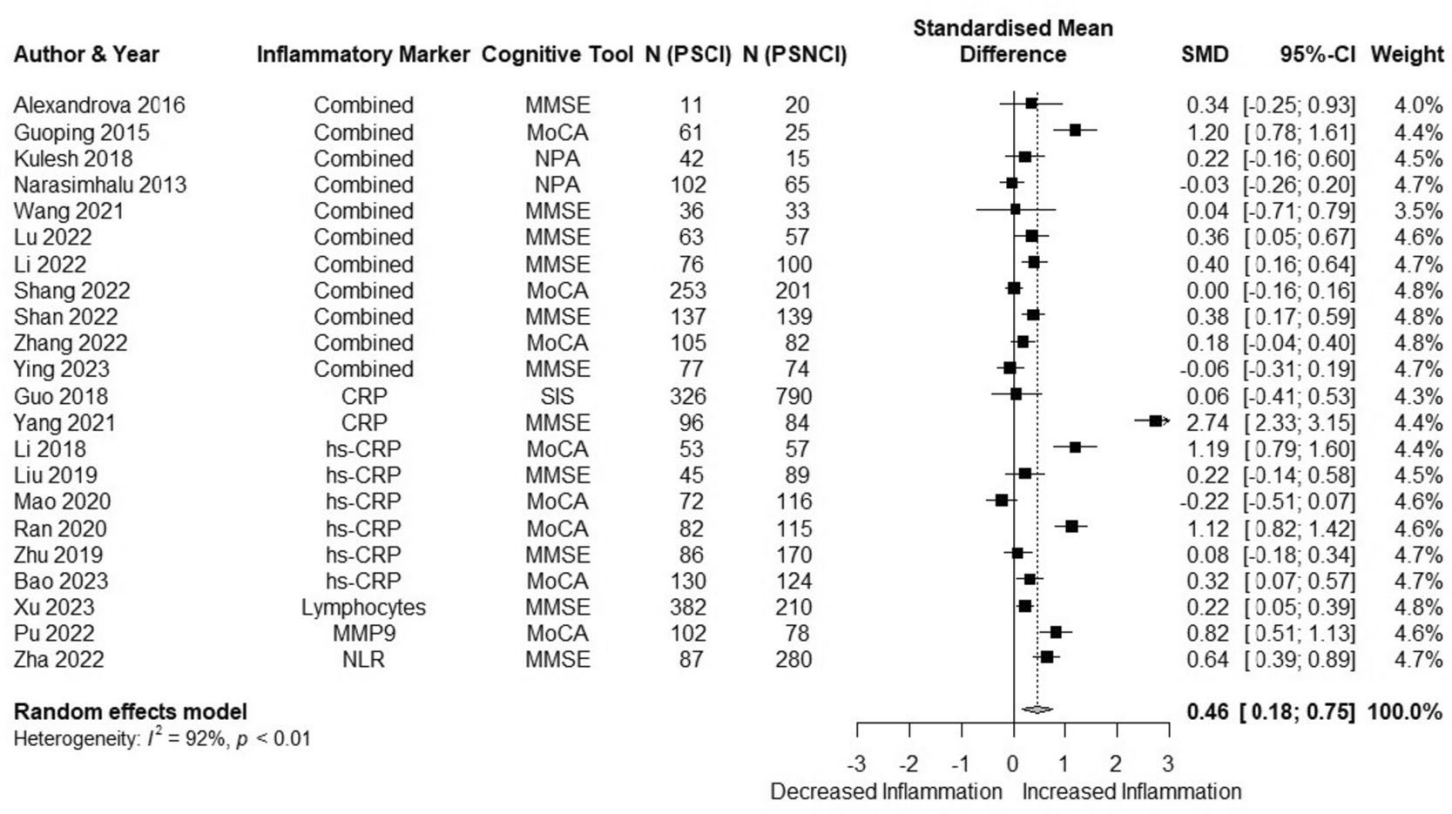
Meta-analysis of human studies: inflammatory parameters in stroke survivors with cognitive impairment compared to those without cognitive impairment. CRP, C-reactive protein; hs-CRP, high sensitive CRP; MMP9, matrix metallo proteinase 9; NLR, neutrophil–lymphocyte ratio; MMSE, mini-mental state examination; MoCA, Montreal cognitive assessment; NPA, neuropsychological assessment; SIS, six-item-screener; PSCI, post-stroke cognitive impairment; PSNCI, post-stroke non cognitive impairment

In total, 22 studies with 50 outcomes were included in this analysis. The meta-analysis using unnested data showed a shift towards a pro-inflammatory state (SMD = 0.34 (95% CI 0.18; 0.50)), with a large heterogeneity (89%). The three-level meta-analysis showed a considerable heterogeneity attributable to within-study heterogeneity. This was confirmed by an ANOVA comparing both models (*χ*^2^ = 16.7, *p* < 0.0001). For this reason, we aggregated multiple within-study outcomes for the primary analysis. Figure 2 shows the results of the three-level meta-analysis (SMD = 0.46 (95% CI 0.18; 0.75), *I*^2^ = 92%). A sensitivity analysis excluding studies with small sample sizes (*n* < 100) was carried out (SMD = 0.47 (95% CI 0.13; 0.81), *I*^2^ = 99%). Inspection of the funnel plot (Supplementary Fig. 1) and Egger’s test showed evidence of publication bias (*p* = 0.03). Meta-regression to check for confounders showed that only the use of MoCA as a screening tool for cognitive impairment influenced the results, leading to an increased observed effect size (adjusted *p*-value = 0.03). Subgroup analysis was performed on inflammatory markers present in at least 3 individual studies (Supplementary Figs. 3–8). Table 1 shows the diffence in individual biomarker concentrations. No significant differences between patients with and without post-stroke cognitive impairment were observed for any of the serum biomarkers.

**Table 1:** Subgroup analyses for differences in inflammatory biomarkers between patients with and without PSCI

Subgroup	Number of studies	*N* (PSCI)	*N* (PSNCI)	*I*^2^	SMD (95% CI)
CRP	12	1311	1905	95%	0.50 (− 0.01; 1.02)
WBC	4	289	274	73%	0.21 (− 0.28; 0.70)
Lymphocytes	4	817	567	84%	− 0.04 (− 0.47; 0.38)
IL-1b	4	281	205	70%	0.20 (− 0.33; 0.74)
IL-6	5	386	287	90%	0.57 (− 0.37; 0.74)
IL-10	3	221	154	63%	0.16 (− 0.55; 0.88)
TNFa	3	220	180	87%	0.26 (− 0.89; 1.41)

*CRP* C-reactive protein, *WBC* white blood cells, *IL* interleukin, *TNFa* tumor necrosis factor alpha

### Correlations Between Level of Inflammation and Cognitive Functioning in Stroke Survivors

**Fig. 3:**
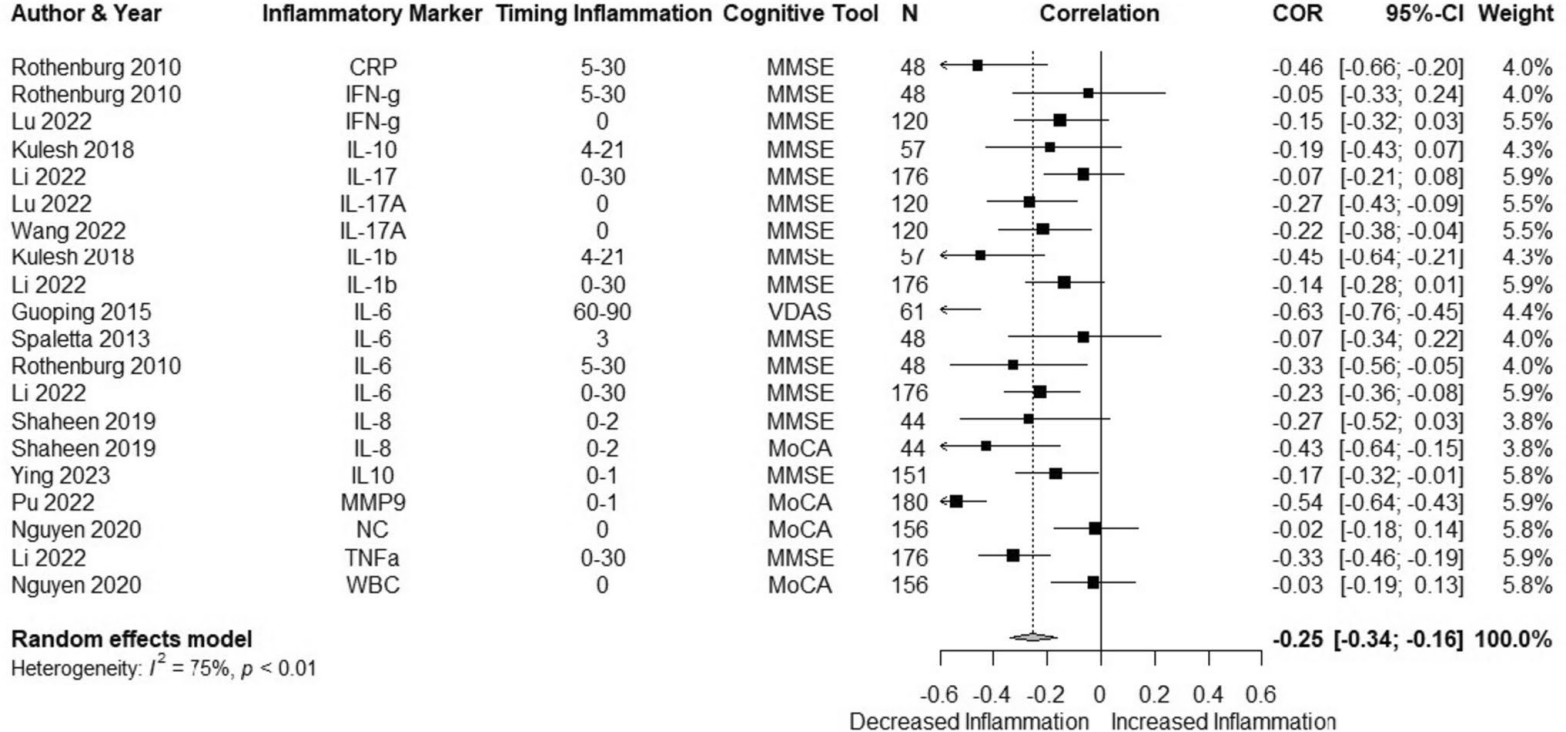
Meta-analysis of human studies: correlations between inflammation and cognition. NC, neutrophil count; IL, interleukin; CRP, C-reactive protein; WBC, white blood cells; IFN-g, interferon-gamma; MMP9, matrix metallo proteinase 9; TNFa, tumor necrosis factor alpha; VDAS, vascular dementia assessment scale; MMSE, mini-mental state examination; MoCA, Montreal cognitive assessment; NPA, neuropsychological assessment

Figure 3 shows the results of the meta-analysis on the correlation between inflammation and cognition after stroke. In total, 11 studies with 20 outcomes were included in the analysis. The meta-analysis using unnested data showed a small negative correlation between level of inflammation and cognitive functioning (*r* =  − 0.25 (95% CI − 0.34; − 0.16)) with large heterogeneity (75%). A sensitivity analysis excluding studies with small sample sizes (*n* < 100) was carried out (*r* =  − 0.20 (95% CI − 0.31; − 0.09), *I*^2^ = 79%). Inspection of the funnel plot (Supplementary Fig. 9) and Egger’s test showed no evidence of publication bias (*p* = 0.3). Multi-level meta-analysis did not show considerable within-study heterogeneity. Meta-regression showed that only the timing of cognitive testing had a small effect on the results; the observed correlation weakened as time between stroke and cognitive testing increased (adjusted *p* = 0.03). Only IL-6 was investigated in at least 3 individual studies (Supplementary Fig. 10). A subgroup analysis did not show a correlation between IL-6 and cognition (*r* =  − 0.33 (95% CI − 0.66; 0.11), *I*^2^ = 79%).

### The Effect of Anti-inflammatory Interventions on Cognitive Functioning in Animal Studies

**Fig. 4:**
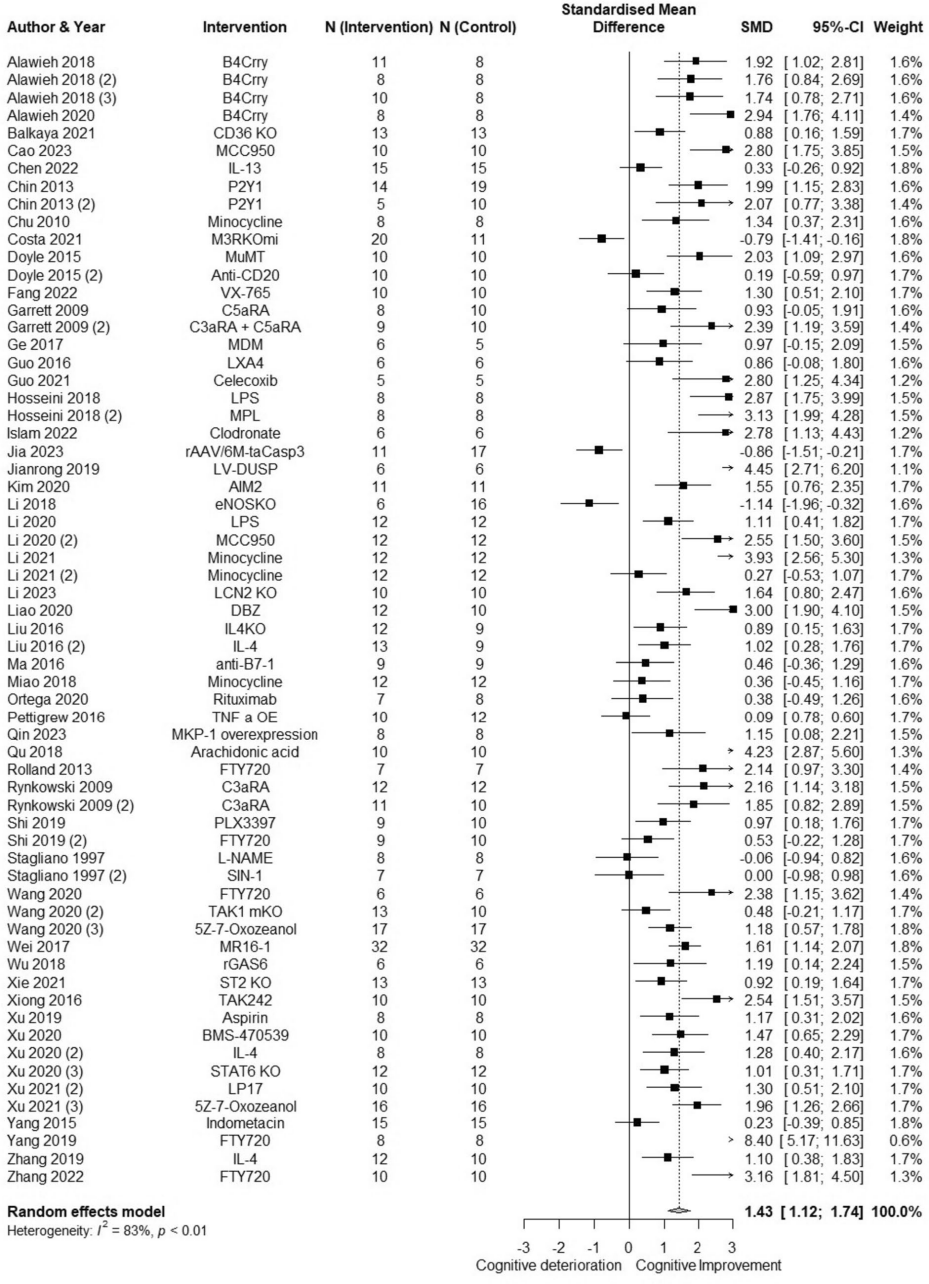
Meta-analysis of animal studies: the effect of anti-inflammatory interventions on cognitive functioning. B4Crry, anti-annexin scFV linked to complement receptor 1–related gene/protein y; CD36, cluster of differentiation 36; P2Y1, P2Y purinoceptor 1; M3RKOmi, microglial muscarinic acetylcholine receptor 3 knockout; MuMT, peripheral B-cell deficient; CD20, cluster of differentiation 20; VX-765, Caspase-1 inhibitor; MKP-1, mitogen-activated protein kinase-1; C5aRA, C5a receptor antagonist; C3aRA, C3a receptor antagonist; MDM, monocyte-derived macrophages; LXA4, lipoxin A4; LPS, lipopolysacaharide; MPL, monophosphoryl lipid A; rAAV-taCasp3, partial depletion of CD11c + microglia; LV-DUSP, lentiviral dual-specificity phosphatase; AIM2, absent in melanoma 2; eNOSKO, endothelial nitric oxide synthase knockout; MCC950, inhibitor of NOD-like receptor protein-3 (NLRP3); DBZ, tanshinol borneol ester; IL-4, interleukin-4; IL-13, interleukin-13; anti-B7-1, monoclonal anti-B7 antibody; TNF-a OE, tumor necrosis factor α overexpression; FTY720, fingolimod; PLX3397, inhibitor of colony stimulating factor 1 receptor (CSF1R); L-NAME, L-N.^G^-Nitro arginine ester; SIN-1, peroxynitrite donor; TAK1 mKO, microglia/macrophage-specific knockout of transforming growth factor-β-activated kinase 1 (TAK1); MR16, anti-interleukin-6 receptor antibody; rGAS6, recombinant growth arrest-specific 6; ST2 KO, interleukin 1 receptor-like 1 knockout; TAK242, selective toll-like receptor 4 inhibitor; BMS-470539, melanocortin MC1 receptor agonist; STAT6 KO, signal transducer and activator of transcription 6 knockout; LP17, inhibitor of triggering receptor expressed on myeloid cells 1 (TREM-1)

We included 50 animal studies, which assessed 175 outcome variables. The analysis using unnested data showed that an intervention targeting inflammation resulted in an improvement in cognition (SMD = 1.37 (95% CI 1.15; 1.60)) with large heterogeneity (82%). The multi-level meta-analysis showed considerable heterogeneity attributable to within-study heterogeneity. This was confirmed by an ANOVA comparing both models (*χ*^2^ = 38.4, *p* < 0.0001). For this reason, we aggregated multiple within-study outcomes for the primary analysis. Figure 4 shows the results of a 3-level meta-analysis (SMD = 1.43 (95% CI 1.12; 1.74), *I*^2^ = 83%). Inspection of the funnel plot (Supplementary Fig. 11) and Egger’s test showed evidence for publication bias (*p* < 0.01). Meta-regression of unnested data showed that only timing of intervention (adjusted *p* < 0.01) affects the results, indicating that the effect of the interventions decreases as time between stroke and intervention increases. Interventions targeting inflammation resulted in a larger improvement in cognition in mice than in rats, but not after Bonferroni correction (adjusted *p* = 0.06). A subgroup analysis was performed for stroke subtypes: hemorrhagic stroke (SMD = 1.51 (95% CI 1.02; 1.99), *I*^2^ = 73%) and ischemic stroke (SMD = 1.35 (95% CI 0.95; 1.77), *I*^2^ = 86%). In both subgroups, timing of intervention was a significant moderator (*p* < 0.01 in both subgroups).

### Subgroup Analyses of Anti-inflammatory Interventions in Animal Studies

We identified nine subgroups of interventions targeting inflammation based on similar inflammatory pathways. Of these, eight were represented in at least three individual studies. We performed a random-effects meta-analysis on these eight subgroups (Supplementary Figs. 12–19) using unnested data. Table 2 shows the summary results of the subgroup analyses. Five out of eight subgroups of interventions showed a positive effect on cognitive functioning. Largest effect sizes were found with the immune suppressor fingolimod (SMD = 2.11 (95% CI 0.75; 3.47), *I*^2^ = 81%) and with complement inhibition (SMD = 1.94 (95% CI 1.50; 2.37), *I*^2^ = 51%). Interventions applying IL-4 showed a large effect size with low heterogeneity (SMD = 1.04 (95% CI 0.85; 1.23), *I*^2^ = 0%), while interventions targeting microglia phenotype (SMD = 1.18 (95% CI 0.76; 1.59), *I*^2^ = 61%) showed a large effect size with moderate heterogeneity. Interventions using general anti-inflammatory interventions showed a large effect size with high heterogeneity (SMD = 1.51 (95% CI 0.89; 2.13), *I*^2^ = 84%), while interventions using B cell depletion (SMD = 0.95 (95% CI − 0.15; 2.06), *I*^2^ = 85%), microglia depletion (SMD = -0.03 (95% CI -0.90; 0.84, I2 = 81%) and minocycline (SMD = 1.37 (95% CI − 0.51; 3.26), *I*^2^ = 85%) showed no effect on cognition.

**Table 2:** Subgroup analyses for the type of anti-inflammatory intervention in animals: effect on cognition

Subgroup	Number of studies	Number of variables	*I*^2^	SMD (95% CI)
General anti-inflammatory	15	43	83%	1.66 (1.14; 2.18)
B cell depletion	3	10	85%	0.95 (− 0.15; 2.06)
Microglia depletion	4	10	81%	− 0.03 (− 0.90; 0.84)
Microglia phenotype	5	14	61%	1.18 (0.76; 1.59)
Complement inhibition	8	19	51%	1.94 (1.50; 2.37)
Fingolimod	5	11	81%	2.11 (0.75; 3.47)
Minocycline	4	5	85%	1.37 (− 0.51; 3.26)
IL-4	4	12	0%	1.04 (0.85; 1.23)

## Discussion

Patients with PSCI have higher serum or CSF levels of inflammatory markers compared to patients without PSCI. In addition, we found a negative correlation between the level of inflammatory markers and level of cognitive (dis)functioning. In animal stroke models, cognitive functioning was better in animals treated with anti-inflammatory interventions compared to controls. In subgroup analyses, five out of eight intervention subgroups showed a positive effect on cognitive functioning.

While most of the subgroups of anti-inflammatory interventions showed a statistically significant effect on animal cognitive performance, none of the individual inflammatory markers were increased in patients with PSCI, suggesting that individual inflammatory markers might not be a good reflection of PSCI. Furthermore, interventions in animals administered < 24 h of stroke showed the largest effect sizes, while the timing of measuring inflammatory markers did not significantly moderate the effect size. This might be caused by the different temporal profiles of the individual inflammatory markers [27].

In a recent large meta-analysis on the relation between inflammation and Alzheimer’s disease (AD), multiple markers of inflammation were increased in AD patients compared to controls, both in peripheral blood and CSF [28]. It is known that increased inflammation can precede the onset of AD and VaD [29]. These data, combined with our finding of a relation between increased inflammatory markers and impaired cognition, suggest that similar processes downstream of inflammatory changes might underlie cognitive impairment in AD, VaD, and PSCI. These include inflammation-mediated blood–brain-barrier dysfunction, synaptic pruning or dysfunction, or neuronal degeneration [30, 31].

In many of our analyses, we observed a high level of heterogeneity, which can have various causes. In human studies, the meta-regression analysis showed that timing and mode of cognitive assessment affected the results; the effect size was highest when using MoCA as a screening tool, while the correlation between inflammation and cognition diminished as time between stroke and cognitive screening increased. Further heterogeneity may be caused by the demographic (age, sex), clinical (history of stroke, stroke severity, comorbidities), or genetic heterogeneity of the population, by differences in study design or laboratory equipment used for testing, or by differences between individual inflammatory markers. Furthermore, the majority of inflammatory markers were measured in serum, while evidence shows that some inflammatory factors are strongly localized in the central nervous system (CNS) [32]. We were not able to compare serum and CSF biomarkers, as only 2 measurements in the same study were performed in CSF. In animal studies, we also observed heterogeneity between studies, but to a lesser degree in studies investigating the effect of interventions modulating microglia phenotype and in studies applying IL-4. The IL-4 studies were homogenous in terms of timing of the administration, the timing of cognitive assessment, and tests used for assessment.

We found large effect sizes in interventions modulating the phenotype of reactive microglia, interventions using complement inhibitors, and in studies in which IL-4 was administered. This finding might be caused by their common effect on microglia. Microglia, the resident immune cells of the central nervous system, are thought to play an important role in the development of cognitive impairment. Under normal physiological conditions, microglia are critical for the maintenance of neural tissue hemostasis and normal neuronal functioning. In response to changes in the CNS milieu, due to, e.g., neural tissue injury, infections, and tumors, microglia become reactive. The phenotypes of reactive microglia are highly context-dependent and range from a pro-inflammatory (called also M1) to an anti-inflammatory or regenerative state (called also M2) [33, 34]. Notably, in recognition of their multiple and context-dependent phenotypes, the field is moving from this and other types of simplified dichotomization of reactive microglia [35]. Indeed, the specific phenotype of reactive microglia depends both on time after the stroke event and on the type of stroke; in ischemic stroke, microglia seem to shift from a more anti-inflammatory and neuroprotective phenotype in the early phase to a more pro-inflammatory phenotype in a later phase, while opposite responses are induced by hemorrhagic stroke [36, 37]. It is generally assumed that microglia with a pro-inflammatory phenotype inhibit repair and recovery, while the anti-inflammatory microglia promote resolution of inflammation and enhance regeneration [38]. A shift in microglia phenotype can be achieved through deletion or inhibition of mitogen-associated protein kinase (MAP3K) and transforming growth factor (TGF)-β-activated kinase 1 (TAK1) [39], either through deletion or inhibition, through triggering receptor expressed on myeloid cells (TREM)-1 [40], or through injection of M2-like monocyte-derived macrophages (MDM) [41]. Furthermore, IL-4 can shift microglia towards M2 phenotype through the JAK1/STAT6 pathway [42], while inhibition of complement activation prevents complement opsonins from tagging neurons for phagocytosis by microglia [43]. Inhibition of signaling through complement receptors C5aR and C3aR reduces inflammatory cell infiltration [44].

We did not find an effect of interventions using minocycline, which is similar to the conflicting results of previous research on the effect of minocycline on microglia function. Some studies found that minocycline promotes M2 polarization and inhibits M1 polarization [45], while other studies found no effect of minocycline on microglia as assessed by their morphology [46].

Finally, a large effect was found in interventions administering fingolimod. Fingolimod acts on sphingosine-1-phosphate receptors (S1PRs) and through this pathway affects microglia, lymphocytes, natural killer cells, dendritic cells, macrophages and neutrophils, and other immune cells [47]. It has anti-inflammatory effects through different modes of action and even exerts memory-preservation properties outside of its effect on inflammation [48].

Our meta-analysis shows that anti-inflammatory interventions may be beneficial in decreasing PSCI. So far, trials in stroke patients investigating the effect of anti-inflammatory interventions mostly focused on IL-1, endothelial selectins, and leukocyte infiltration, but did not have cognition as an outcome measure. Minocycline has shown a good safety profile in a small dose-escalation trial [49], while interventions using fingolimod showed a good safety profile and an improvement in modified Rankin scale (mRS), National Institutes of Health Stroke Scale (NIHSS), and infarct growth in initial trials [50]. Currently, a phase II trial on the effect of fingolimod in ischemic stroke and a phase II trial assessing the efficacy of complement inhibition after hemorrhagic stroke are ongoing [51, 52].

The results of our meta-analysis of animal studies indicate that the timing of anti- inflammatory interventions is important with the largest effect being observed when treatment is initiated in the first 24 h after stroke induction. Reduced recruitment of inflammatory cells from the systemic circulation is conceivably one of the underlying mechanisms. Neutrophil infiltration in the brain parenchyma occurs in the acute phase after human stroke [53]. In animal models of stroke, the levels of neutrophils in the peripheral blood rise within 12 h, peaking at 24-h post stroke, and drop to control levels by 48 h [54]. Multiple cytokines and other inflammatory signals are involved in neutrophil activation and recruitment to the brain parenchyma after stroke and have been evaluated as stroke treatments [55].

Some limitations need to be acknowledged when interpreting the results of our meta-analysis. First, we observed a high level of heterogeneity in many of our analyses, especially in human studies, which we were unable to fully explain. Second, in an ideal situation, multiple regressions need to be used to identify how different populations, outcomes, or study-related factors can influence our results. However, given the limited sample sizes and information reported in the included studies, this was not feasible. Third, we found evidence of publication bias, which may have led to an overestimation of the effect sizes. Finally, the majority of human studies investigated peripheral inflammation only at a single time point. Collecting inflammatory markers longitudinally from CSF would resolve temporal and spatial variability in inflammatory responses [56]. Unfortunately, this implies practical difficulties and invasive measurements. The strengths of our study are the extensive overview of the literature and inclusion of animal studies.

Our study has implications for future studies. First, definitions and outcome reporting should be homogenized. In the identified studies, the MoCA was the preferred tool for defining PSCI. Second, as temporal differences in inflammatory markers are common, it is important to report on time delay between stroke and sample collection. Preferably, longitudinal studies with multiple sample collections should be used to investigate the inflammatory response over time in patients with PSCI. Last, future studies on anti-inflammatory interventions preventing PSCI should focus on complement inhibition, fingolimod or IL-4 while attempting to minimize time delay between stroke onset and administration. Safety of IL-4, fingolimod and complement inhibition should first be assessed by phase I or II trials, which are currently on their way.

## Supplementary Information

Below is the link to the electronic supplementary material.

Supplementary file1 (PDF 340 KB)

Supplementary file2 (DOCX 680 KB)

Supplementary file3 (DOCX 154 KB)

## Data Availability

All data supporting the findings of this study are available within the paper and its Supplementary Information. Supplementary Tables 1 and 2 contain included data of human studies. Data from animal studies can be found in the references of supplementary Table 3.
