# Supplementary material for: Inflammation, Anti-inflammatory Interventions, and Post-stroke Cognitive Impairment: a Systematic Review and Meta-analysis of Human and Animal Studies
Source: Transl Stroke Res. 2023 Nov 28;16(2):535–46. doi: 10.1007/s12975-023-01218-5 (PMC11976800; doi:10.1007/s12975-023-01218-5)
Supplement: Supplementary file 1 — Supplementary file1 (PDF 340 KB) [file 12975_2023_1218_MOESM1_ESM.pdf]

Search syntax

(stroke[MeSH Terms] OR stroke*[Title/Abstract] OR Intracranial Hemorrhage [MeSH Terms] OR Intracranial Hemorrhage* [Title/Abstract] OR Cerebral Hemorrhage[MeSH Terms] OR Cerebral Hemorrhage*[Title/Abstract] OR Subarachnoid Hemorrhage [Mesh] OR Subarachnoid Hemorrhage* [Title/Abstract] OR Intracranial Haemorrhage [MeSH Terms] OR Intracranial Haemorrhage* [Title/Abstract] OR Cerebral Haemorrhage[MeSH Terms] OR Cerebral Hemorrhage*[Title/Abstract] OR Subarachnoid Haemorrhage [Mesh] OR Subarachnoid Haemorrhage* [Title/Abstract] OR SAH [Title/Abstract]OR cerebrovascular accident [Title/Abstract] OR CVA [Title/Abstract] OR Cerebral Infarction [MeSH Terms] OR Cerebral Infarction* [Title/Abstract] OR Brain Ischemia [MeSH Terms] OR Brain Ischemia [Title/Abstract] OR Infarction, Middle Cerebral Artery [MeSH Terms] OR Middle cerebral artery occlusion* [Title/Abstract] OR MCAO [Title/Abstract] OR Hypoxia-Ischemia, Brain[MeSH Terms] OR Hypoxia-Ischemia [Title/Abstract]) AND (Complement System Proteins [MeSH Terms] OR Complement System*[Title/Abstract] OR complement protein*[Title/Abstract] OR Complement Activation[MeSH Terms] OR Complement Activation*[Title/Abstract] OR Complement Pathway, Classical[MeSH Terms] OR complement pathway, alternative[MeSH Terms] OR Classical Complement Pathway*[Title/Abstract] OR Alternative Complement Pathway*[Title/Abstract] OR Complement C1q[MeSH Terms] OR Complement C3[MeSH Terms] OR Complement C5[MeSH Terms] OR Complement C5a [MeSH Terms] OR c1q[Title/Abstract] OR c3[Title/Abstract] OR c5[Title/Abstract] OR inflammation[MeSH Terms] OR Inflammation*[Title/Abstract] OR inflammasomes [MeSH Terms] OR inflammasom*[Title/Abstract] OR inflammat*[Title/Abstract] OR gliosis[MeSH Terms] OR gliosis[Title/Abstract] OR astrogliosis [Title/Abstract] OR Neuroglia[MeSH Terms] OR glia*[Title/Abstract] OR astrocyt*[Title/Abstract] OR microgl*[Title/Abstract] OR neuroglia*[Title/Abstract] OR GFAP*[Title/Abstract] OR iba1*[Title/Abstract] OR Immune System[MeSH Terms] OR Immune System*[Title/Abstract] OR T-Lymphocytes[Mesh] OR B-Lymphocyte*[Title/Abstract] OR TLymphocyte*[Title/Abstract] OR B-cell*[Title/Abstract] OR T-cell* Title/Abstract] OR "t cell*"[Title/Abstract] OR "B cell*"[Title/Abstract] OR Macrophages[MeSH Terms] OR Macrophages[Title/Abstract] OR Macrophag*[Title/Abstract] OR Reactive Oxygen Species[MeSH Terms] OR Reactive Oxygen Species[Title/Abstract] OR ros[Title/Abstract] OR Interleukin-1beta[Title/Abstract] OR IL-1 b*[Title/Abstract] OR catabolin[Title/Abstract] OR IL-1b*[Title/Abstract] OR Interleukins[MeSH Terms] OR Interleukin*[Title/Abstract] OR Interleukin-6[MeSH Terms] OR Interleukin-6[Title/Abstract] OR IL-6[Title/Abstract] OR IL6[Title/Abstract] OR Interleukin-4[MeSH Terms] OR Interleukin-4[Title/Abstract] OR IL-4[Title/Abstract] OR IL4[Title/Abstract] OR Interleukin-8[MeSH Terms] OR Interleukin-8[Title/Abstract] OR IL-8[Title/Abstract] OR IL8[Title/Abstract] OR Interleukin-10[MeSH Terms] OR Interleukin-10[Title/Abstract] OR IL10[Title/Abstract] OR IL10[Title/Abstract] OR Interleukin-13[MeSH Terms] OR Interleukin-13[Title/Abstract] OR IL13[Title/Abstract] OR IL-13[Title/Abstract] OR Interleukin-17[MeSH Terms] OR Interleukin17[Title/Abstract] OR IL17[Title/Abstract] OR IL-17[Title/Abstract] OR IL17A[Title/Abstract] OR IL17F [Title/Abstract] OR IL-17A[Title/Abstract] OR IL-17F[Title/Abstract] OR CTLA8 [Title/Abstract] OR CTLA-8 [Title/Abstract] OR alarmins [MeSH Terms] OR alarmin*[Title/Abstract] OR damage associated molecular patterns [Title/Abstract] OR DAMPS[Title/Abstract] OR Tumor Necrosis Factor alpha[MeSH Terms] OR Tumor Necrosis Factor-alpha[Title/Abstract] OR Tumor Necrosis Factor-a* [Title/Abstract]

OR TNFA[Title/Abstract] OR TNF-a*[Title/Abstract] OR Interferon-gamma [MeSH Terms] OR Interferon-g*[Title/Abstract] OR immune interferon[Title/Abstract] OR IFNG[Title/Abstract] OR Matrix Metalloproteinase 9[MeSH Terms] OR Matrix Metalloproteinase 9[Title/Abstract] OR Metalloproteinase 9[Title/Abstract] OR MMP9[Title/Abstract] OR mmp9[Title/Abstract] OR Chemokine CCL2[MeSH Terms] OR CCL2 [Title/Abstract] OR MCP-1 [Title/Abstract] OR MCP1 [Title/Abstract] OR Chemokine CCL5[MeSH Terms] OR CCL5 [Title/Abstract] OR RANTES [Title/Abstract] OR MIP1a [Title/Abstract] OR MIP1b [Title/Abstract] OR MIP1-a[Title/Abstract] OR Transforming Growth Factor beta[MeSH Terms] OR TGF-B*[Title/Abstract] OR TGFb*[Title/Abstract] OR Chemokine CXCL12[MeSH Terms] OR CXCL12[Title/Abstract] OR SDF1[Title/Abstract] OR SDF-1[Title/Abstract] OR Stromal Cell Derived Factor 1[Title/Abstract] OR Vascular Endothelial Growth Factor A[MeSH Terms] OR Vascular Endothelial Growth Factor B[MeSH Terms] OR Vascular Endothelial Growth Factor[Title/Abstract] OR VEGF*[Title/Abstract] OR InsulinLike Growth Factor I[MeSH Terms] OR Insulin Like Growth Factor*[Title/Abstract] OR IGF*[Title/Abstract] OR Fibroblast Growth Factor 2[MeSH Terms] OR Fibroblast Growth Factor 2[Title/Abstract] OR FGF2[Title/Abstract] OR FGF-2[Title/Abstract] OR FGF 2[Title/Abstract] OR Basic Fibroblast Growth Factor[Title/Abstract] OR Heparin Binding Growth Factor Class II[Title/Abstract] OR HBGF 2[Title/Abstract] OR C-Reactive Protein[MeSH Terms] OR C Reactive Protein*[Title/Abstract] OR CRP [Title/Abstract] OR Leukocyte Count[MeSH Terms] OR Leukocyte Count[Title/Abstract] OR blood cell count[Title/Abstract] OR WBC[Title/Abstract] OR Cyclooxygenase 2[MeSH Terms] OR Cyclooxygenase 2[Title/Abstract] OR Cyclo Oxygenase II[Title/Abstract] OR COX 2[Title/Abstract] OR COX2[Title/Abstract] OR PTGS2[Title/Abstract] OR Prostaglandin Synthase[Title/Abstract] OR Hypoxia-Inducible Factor 1[MeSH Terms] OR Hypoxia Inducible Factor[Title/Abstract] OR HIF 1[Title/Abstract] OR HIF1[Title/Abstract] OR p-Stat3[Title/Abstract] OR phospho stat3[Title/Abstract] OR Nitric Oxide Synthase Type II[MeSH Terms] OR Nitric Oxide Synthase[Title/Abstract] OR iNOS[Title/Abstract] OR inducible NOS[Title/Abstract] OR mTOR [Title/Abstract] OR FDG [Title/Abstract] OR choline [Title/Abstract] OR TSPO [Title/Abstract] OR SSTR [Title/Abstract] OR CB2R [Title/Abstract] OR FPR [Title/Abstract] OR rituximab [Title/Abstract] OR COX [Title/Abstract] OR IL-2 [Title/Abstract] OR integrin [Title/Abstract] OR VAP-1 [Title/Abstract] OR VCAM-1 [Title/Abstract] OR vessel permeability [Title/Abstract] OR fibrinogen [Title/Abstract]) AND (Cognition [MeSH Terms] OR cognit*[Title/Abstract] OR Neurocognitive Disorder[MeSH Terms] OR cognition disorder [MeSH Terms] OR cognit* disorder* [Title/Abstract] OR cognitive dysfunction[MeSH Terms] OR cognit* dysfunction* [Title/Abstract] OR cognit* impairment* [Title/Abstract] OR Neuropsychological Test*[MeSH Terms] OR Neuropsychological Test*[Title/Abstract] OR Mental Status and Dementia Tests[MeSH Terms] OR memory[MeSH Terms] OR attention[MeSH Terms] OR executive function[MeSH Terms] OR language[MeSH Terms] OR Psychomotor Performance[MeSH Terms] OR Psychomotor disorders[MeSH Terms] OR dementia [MeSH Terms] OR memory[Title/Abstract] OR attention[Title/Abstract] OR executive funct*[Title/Abstract] OR executive control* [Title/Abstract] OR language*[Title/Abstract] OR Psychomotor Performance*[Title/Abstract] OR Psychomotor disorder*[Title/Abstract] OR dement*[Title/Abstract] OR novel object recognition [Title/Abstract] OR contextual fear condition* [Title/Abstract] OR cue fear condition* [Title/Abstract] OR Y-maze [Title/Abstract] OR spontaneous alternation [Title/Abstract] OR 5 Choice Serial Reaction Time [Title/Abstract] OR 5CSRTT [Title/Abstract] OR open field [Title/Abstract] OR Light dark box [Title/Abstract] OR Elevated plus maze [Title/Abstract] OR Novelty suppressed feeding

[Title/Abstract] OR Hindlimb Suspension [MeSH Terms] OR tail suspension [Title/Abstract] OR Forced swim [Title/Abstract] OR Rotarod Performance Test [MeSH Terms] OR Rotarod [Title/Abstract] OR Accelerating rotarod [Title/Abstract] OR maze learning [MeSH Terms] OR Morris water maze [Title/Abstract] OR Water maze [Title/Abstract] OR Cylinder test [Title/Abstract])
