# Supplementary material for: Inflammation, Anti-inflammatory Interventions, and Post-stroke Cognitive Impairment: a Systematic Review and Meta-analysis of Human and Animal Studies
Source: Transl Stroke Res. 2023 Nov 28;16(2):535–46. doi: 10.1007/s12975-023-01218-5 (PMC11976800; doi:10.1007/s12975-023-01218-5)

**Supplementary figure 1. Publication bias of Inflammatory markers**


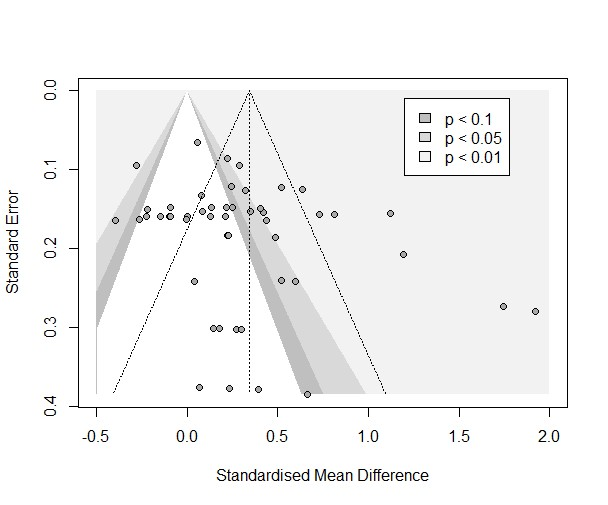


**Supplementary figure 2. Difference in CRP between PSCI and PSNCI**


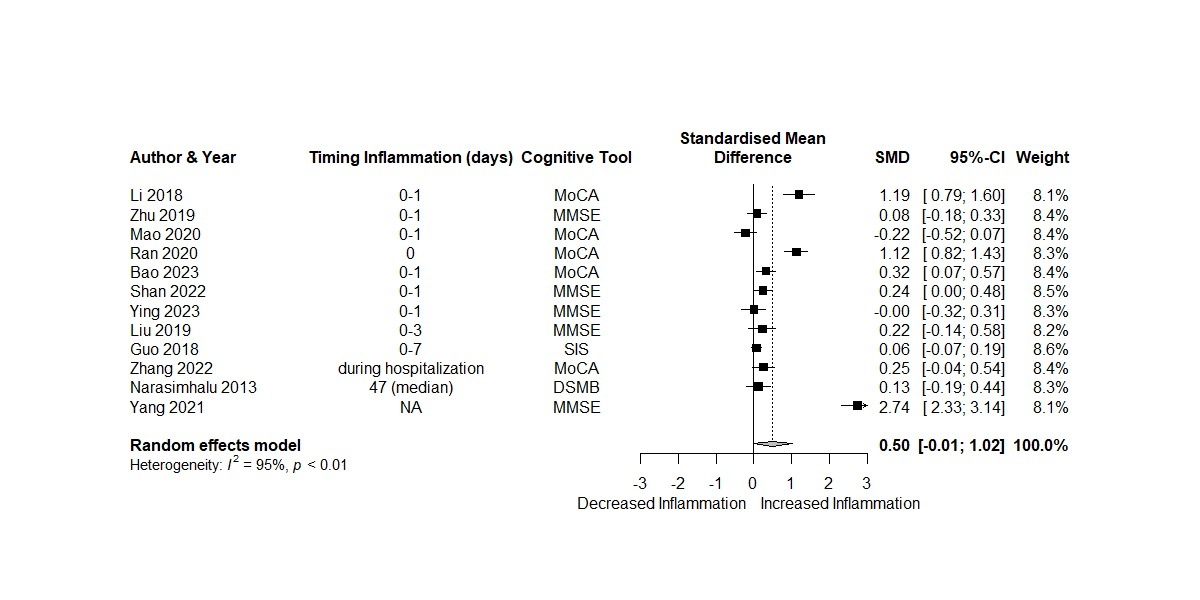


**Supplementary figure 3. Difference in WBC between PSCI and PSNCI**


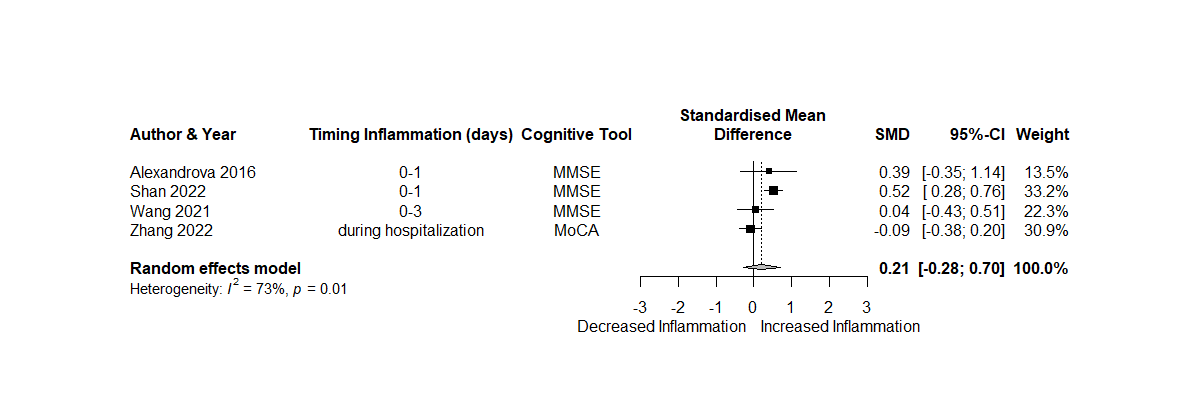


**Supplementary figure 4. Difference in Lymphocytes between PSCI and PSNCI**


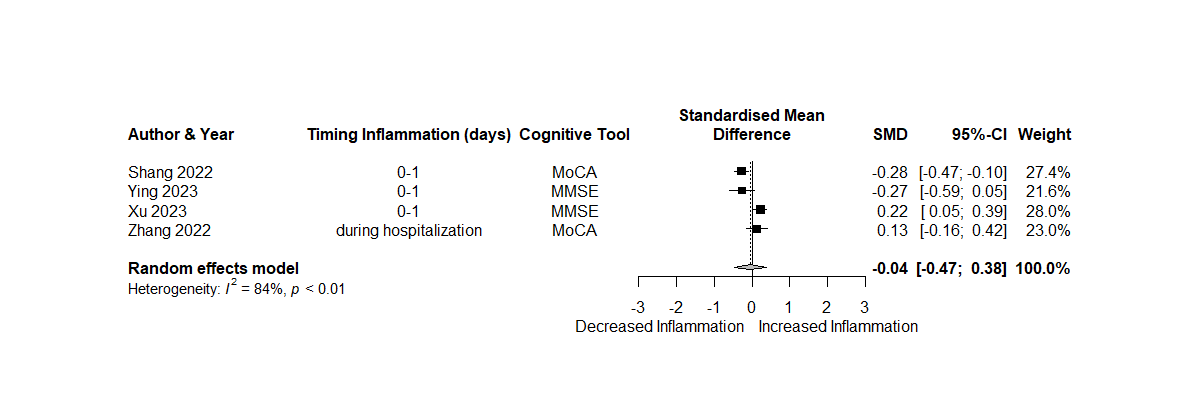


**Supplementary figure 5. Difference in IL-1b between PSCI and PSNCI**


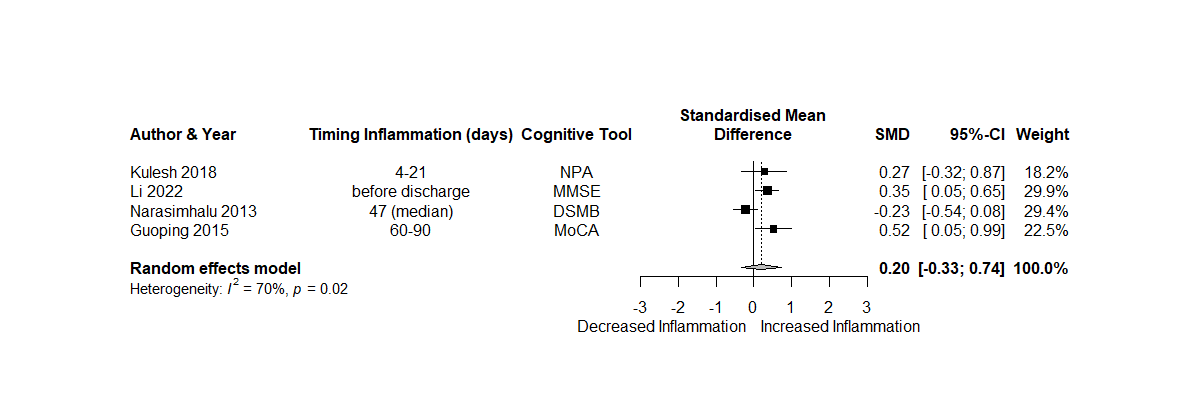


**Supplementary figure 6. Difference in IL-6 between PSCI and PSNCI**


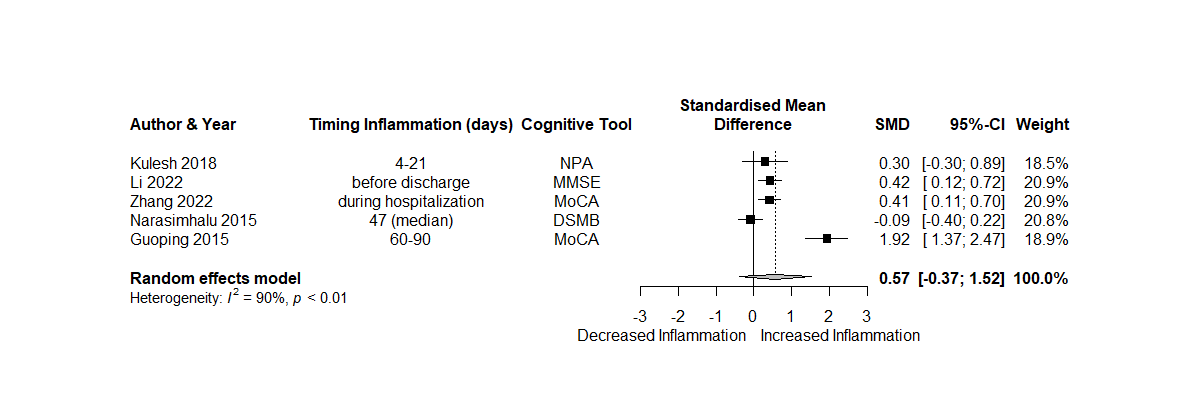


**Supplementary figure 7. Difference in IL-10 between PSCI and PSNCI**


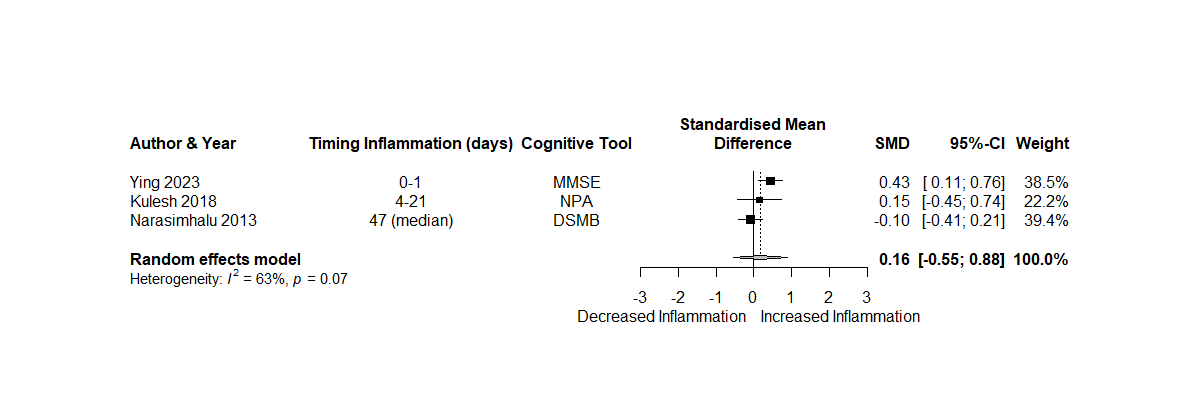


**Supplementary figure 8. Difference in TNFa between PSCI and PSNCI**


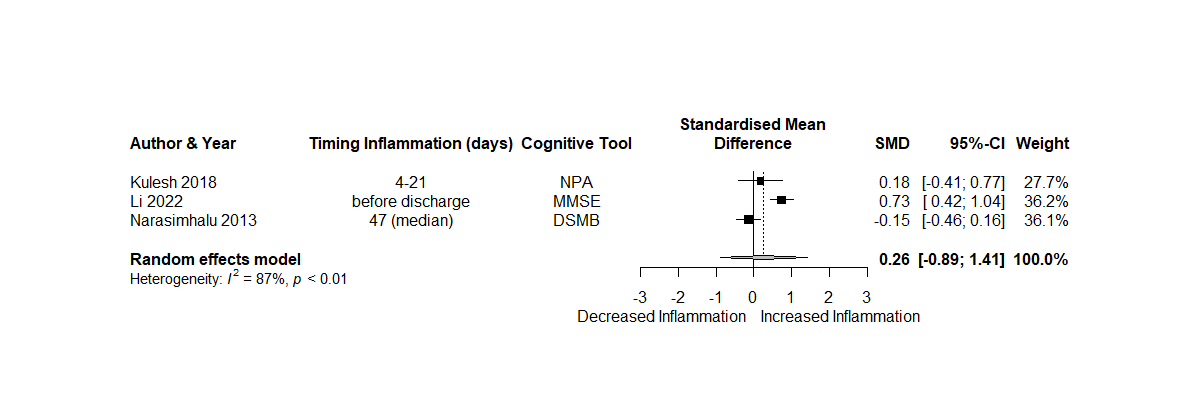


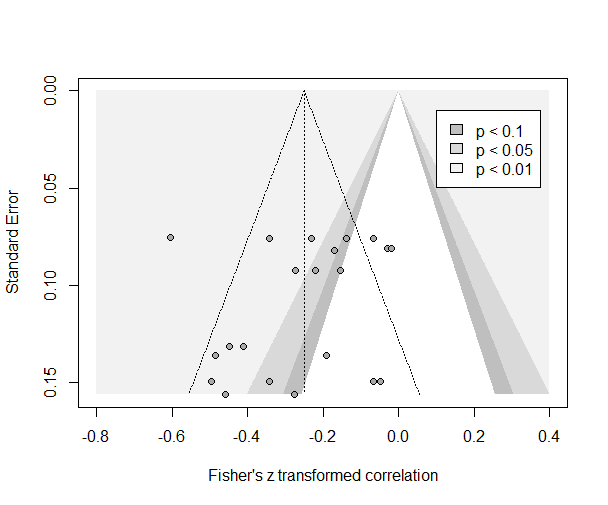
**Supplementary figure 9. Publication bias of correlation between inflammation and cognition**

**Supplementary figure 10. Correlation between IL-6 and cognition**


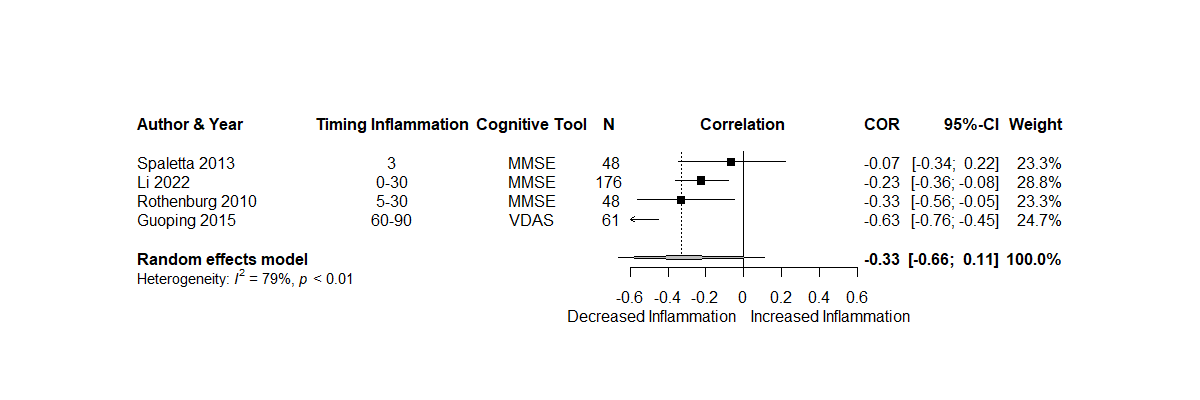


**Supplementary figure 11. Publication bias of animal studies**


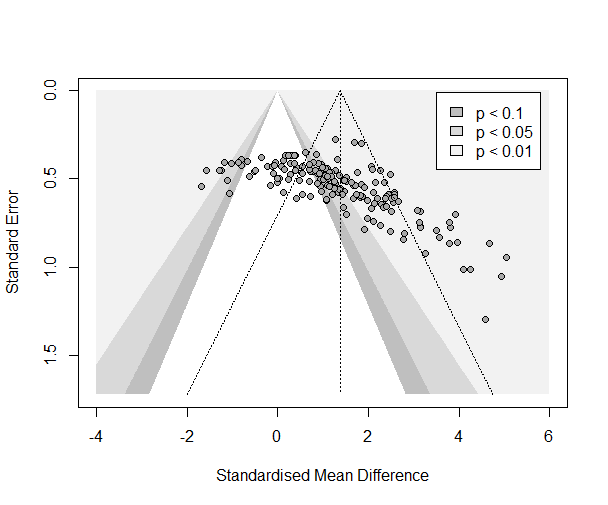


**Supplementary figure 12. Subgroup analysis of General Anti-Inflammatory Interventions**


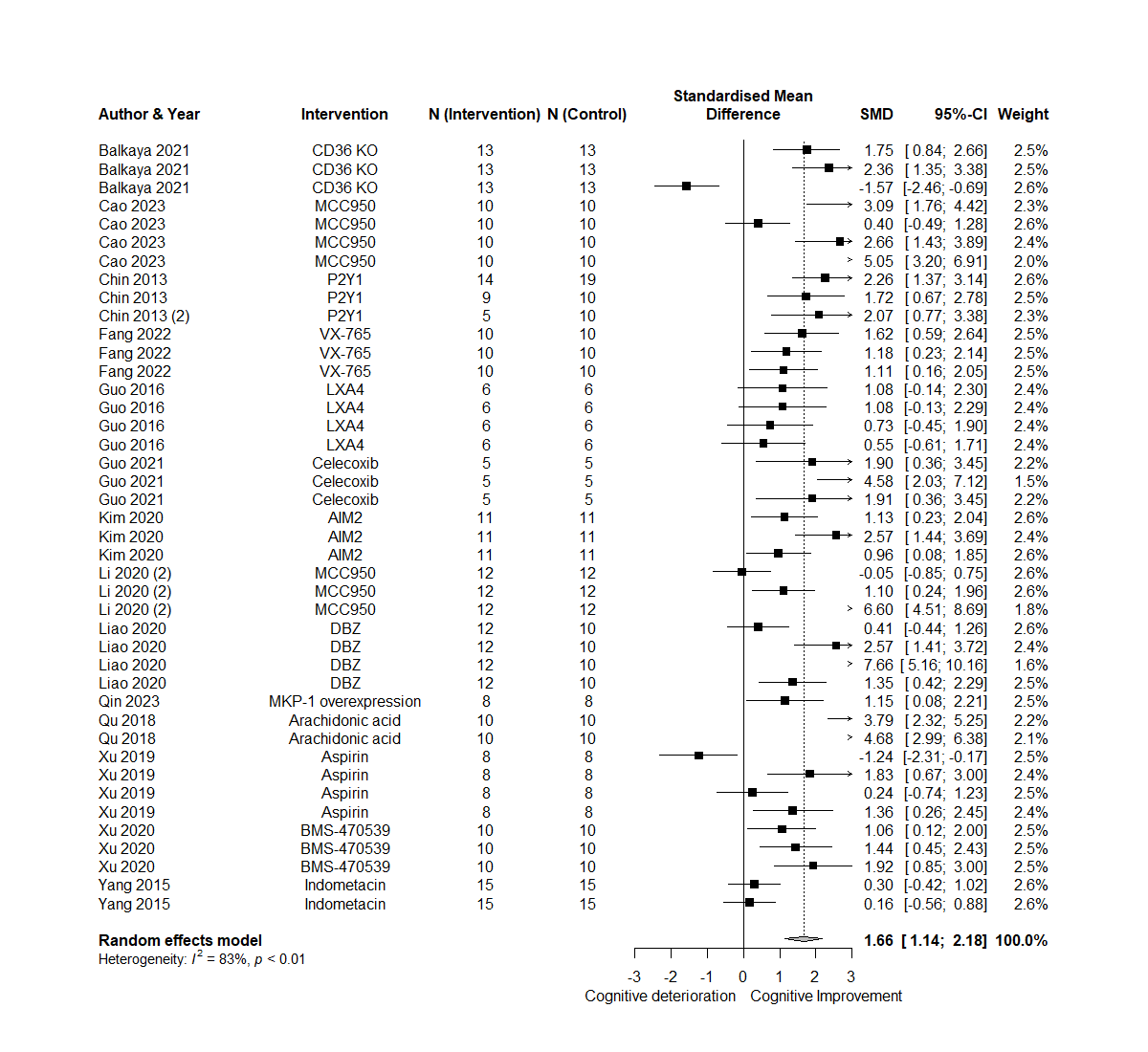


**Supplementary figure 13. Subgroup analysis of B-cell depletion interventions**


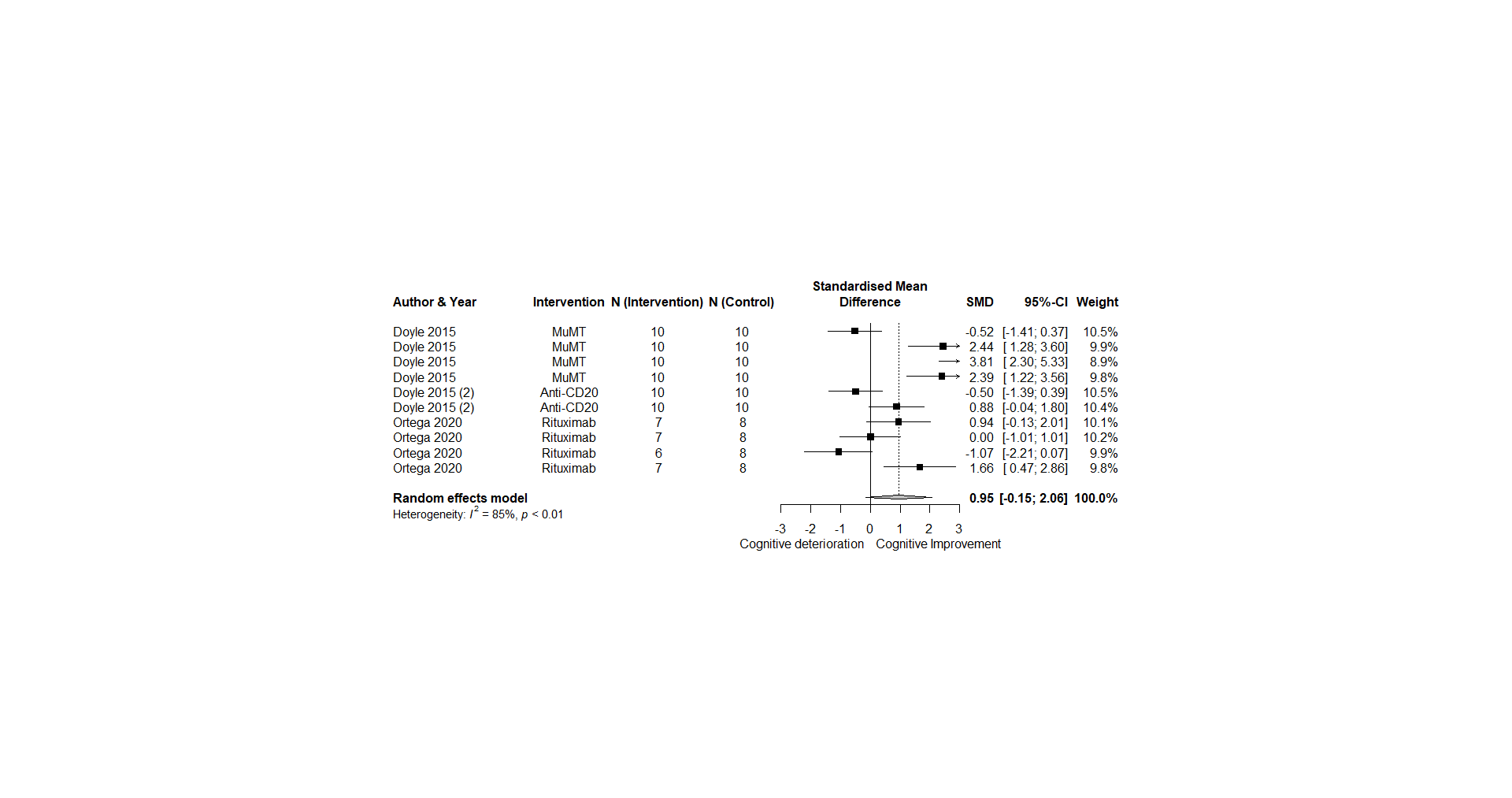


**Supplementary figure 14. Subgroup analysis of Microglia Depletion Interventions**


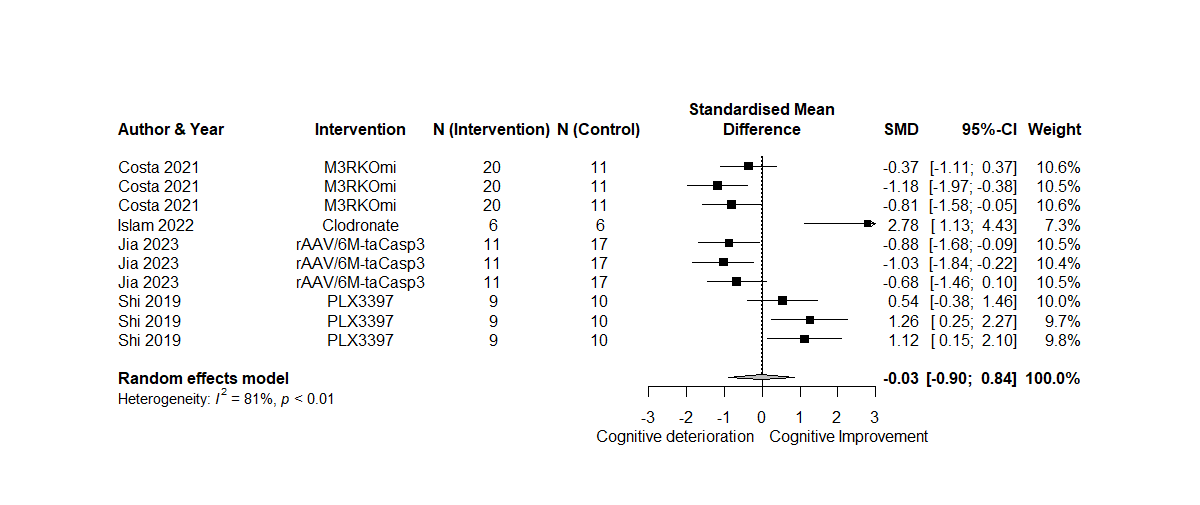


**Supplementary figure 15. Subgroup analysis of Microglia Phenotype Interventions**


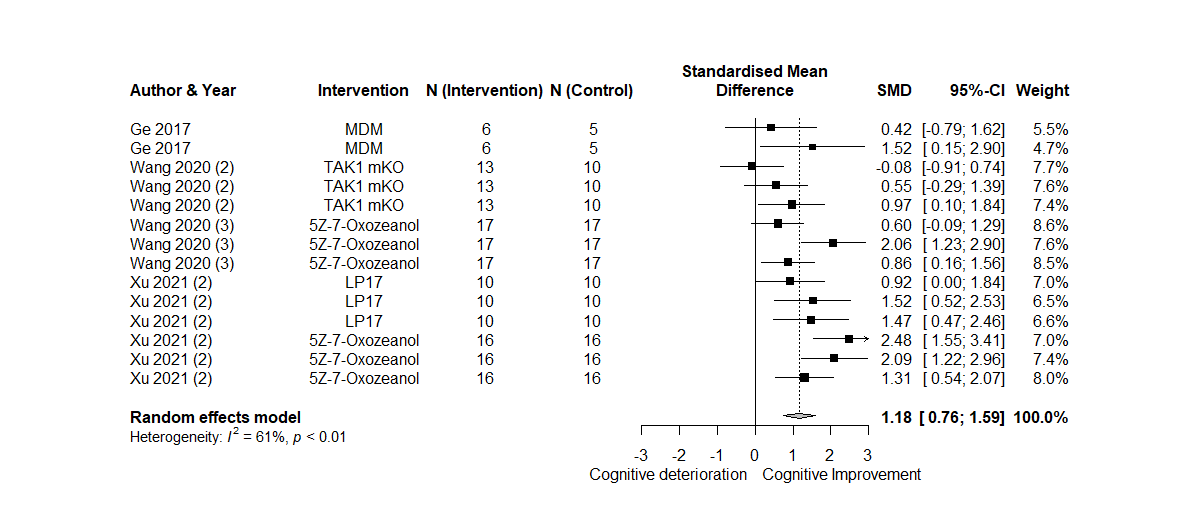


**Supplementary figure 16. Subgroup analysis of Complement Inhibition Interventions**
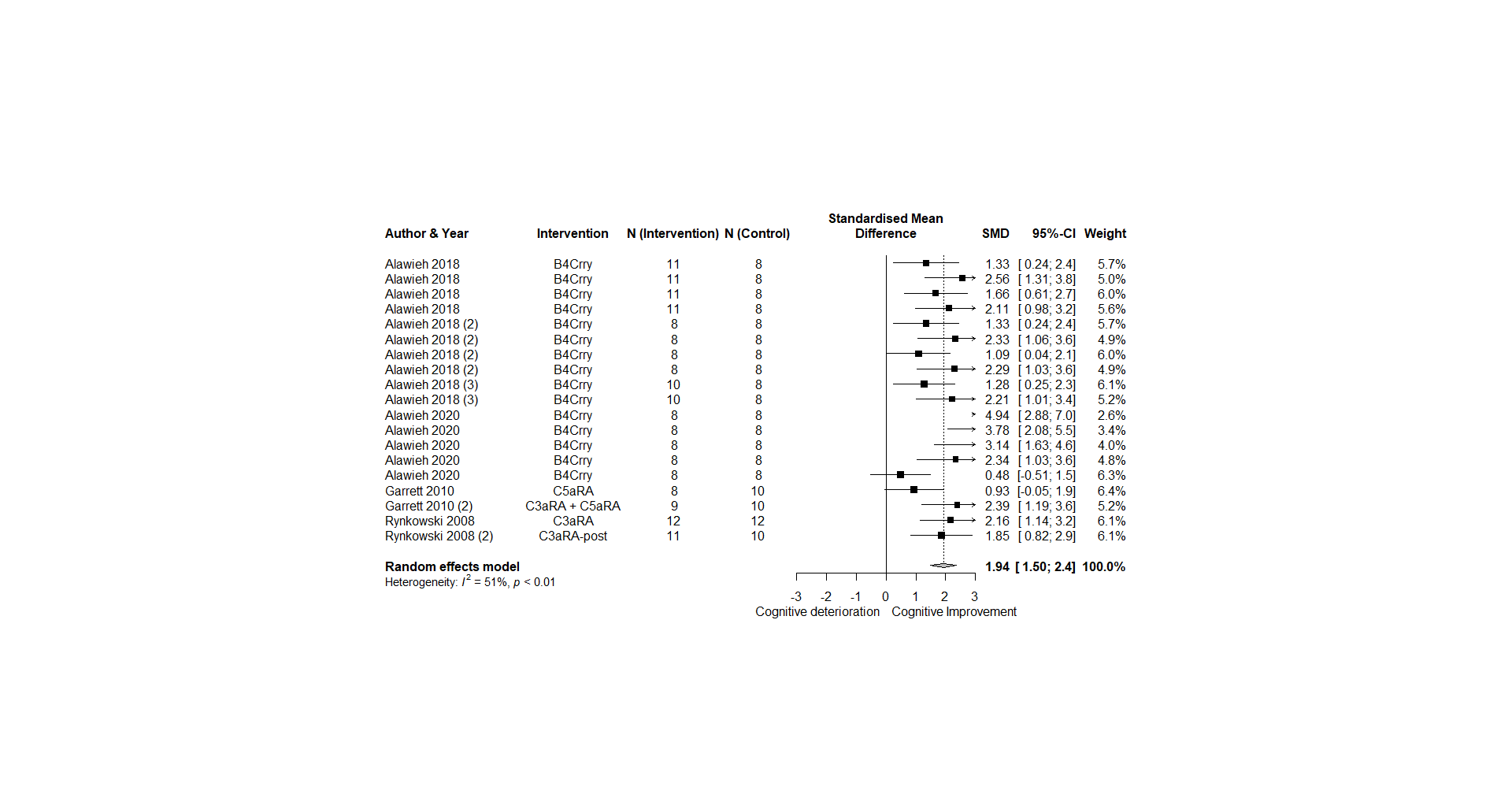


**Supplementary figure 17. Subgroup analysis of Fingolimod Interventions**


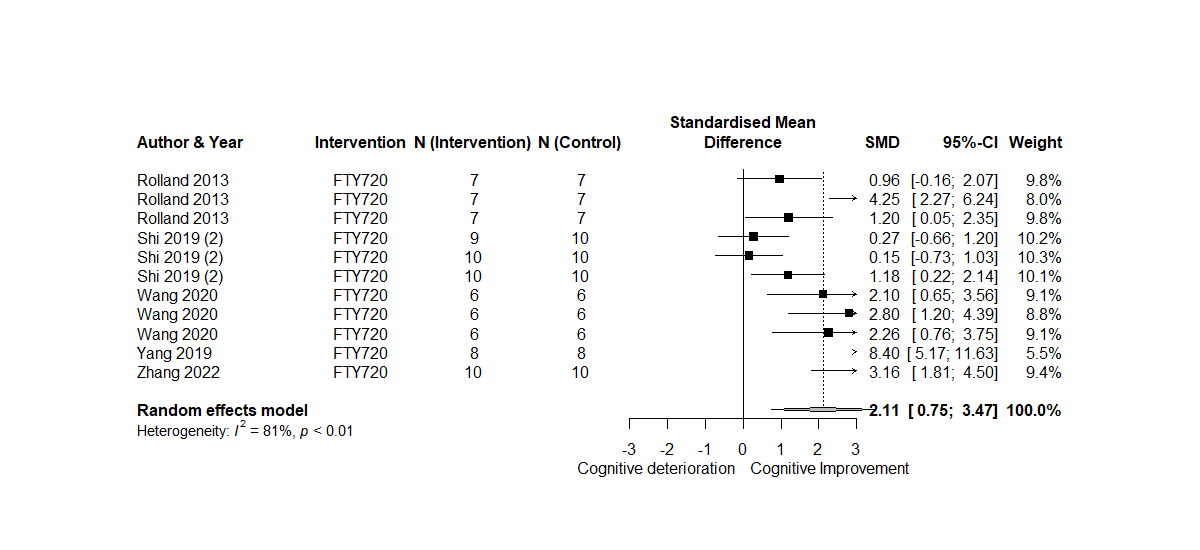


**Supplementary figure 18. Subgroup analysis of Minocycline Interventions**


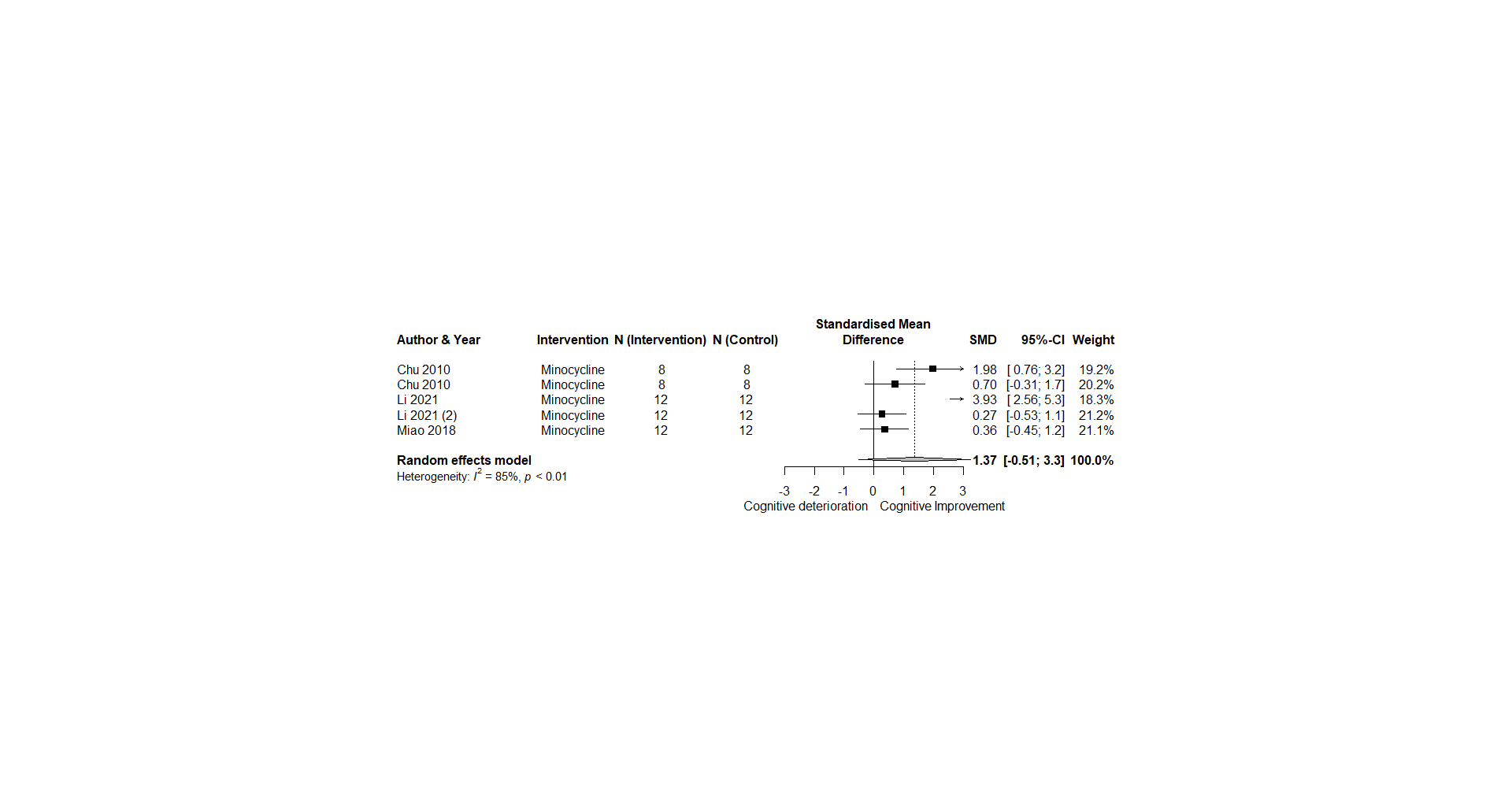


**Supplementary figure 19. Subgroup analysis of IL-4 Interventions**


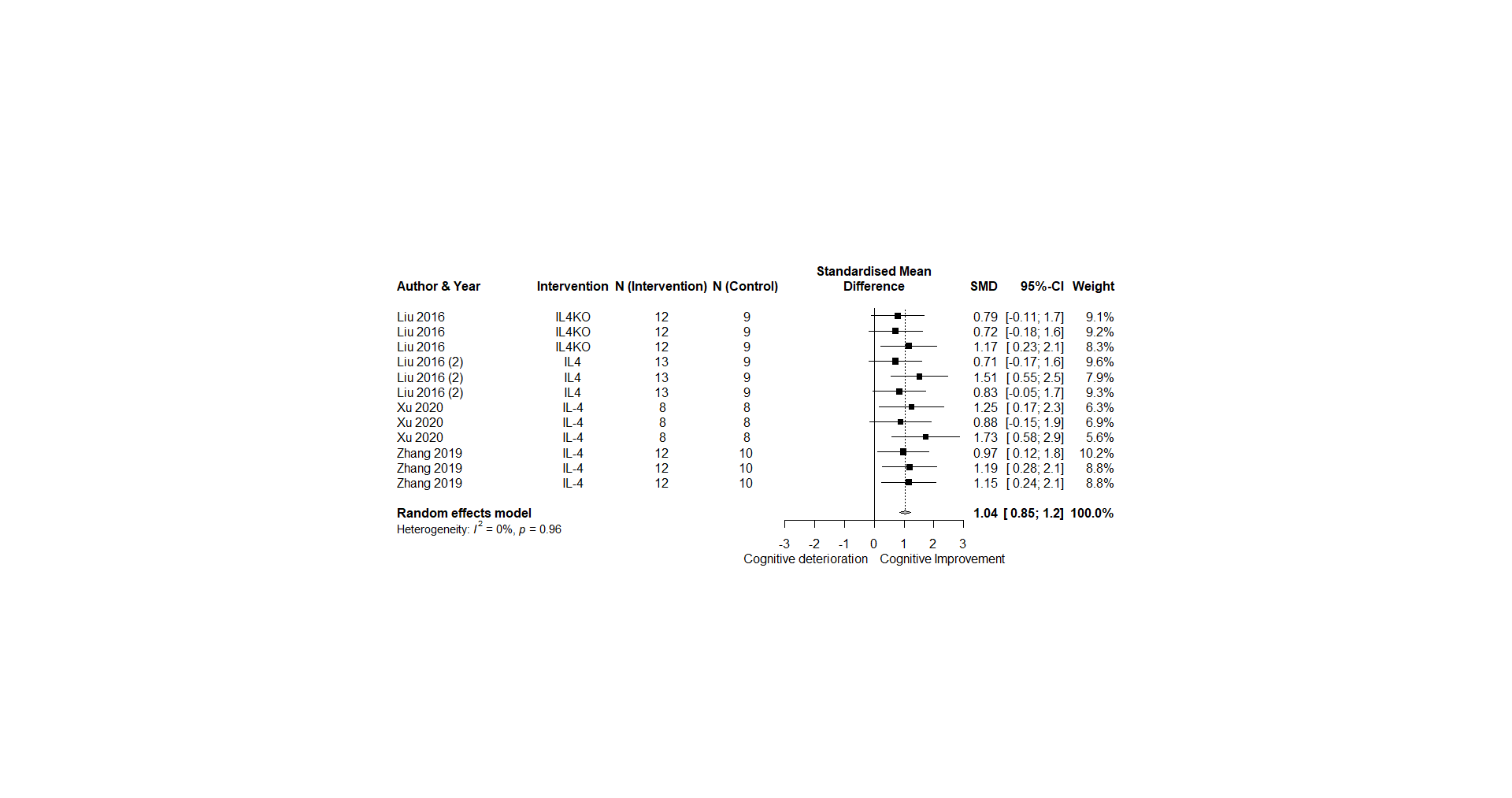

Supplement: Supplementary file 2 — Supplementary file2 (DOCX 680 KB) [file 12975_2023_1218_MOESM2_ESM.docx]
