# Supplementary material for: Inflammation, Anti-inflammatory Interventions, and Post-stroke Cognitive Impairment: a Systematic Review and Meta-analysis of Human and Animal Studies
Source: Transl Stroke Res. 2023 Nov 28;16(2):535–46. doi: 10.1007/s12975-023-01218-5 (PMC11976800; doi:10.1007/s12975-023-01218-5)
Supplement: Supplementary file 3 — Supplementary file3 (DOCX 154 KB) [file 12975_2023_1218_MOESM3_ESM.docx]

**Supplementary table 1. Characteristics of included human studies on inflammatory concentrations of PSCI vs PSNCI patients**

| **Author** | **Stroke** | **Evaluation Cognitive Impairment** | **Timing cognitive evaluation post-stroke** | **PSCI (n)** | **PSNCI (n)** | **Inflammatory biomarker (concentration measure)** | **Biomarker obtained from** | **Timing Inflammatory biomarker post-stroke** | **PSCI**  **mean (SD)** | **PSNCI**  **mean (SD)** |
| --- | --- | --- | --- | --- | --- | --- | --- | --- | --- | --- |
| Li 2018[1] | I | MoCA < 26 | 3 months | 53 | 57 | hsCRP (mg/L) | Serum | Admission | 13 (5) | 19 (5) |
| Zhu 2020[2] | I | MMSE <27 | 1 year | 86 | 170 | hsCRP (mg/L) | Serum | Admission | 6.6 (5.13)* | 4.2 (3.89)* |
| Mao 2020[3] | I | MoCA < 26 | < 1 week | 72 | 116 | hsCRP (mg/L) | Serum | Admission | 4.8 (4.6) | 6.2 (7.1) |
| Ran 2020[4] | I | MoCA < 26 | Admission | 82 | 115 | hsCRP (mg/L) | Serum | Admission | 10.7 (5.3) | 6.2 (2.7) |
| Liu 2019[5] | I | MMSE < 24 | 3 months | 45 | 89 | hsCRP (mg/L) | Serum | As soon as possible | 8.18 (13.18)* | 5.82 (9.01)* |
| Guo 2018 [6] | I | SIS < 5 | 6 months | 326 | 790 | CRP (mg/L) | Serum | < 1 week | 21.4 (17.2) | 20.4 (17.6) |
| Yang 2013 [7] | I | MMSE <25 | < 3 months | 96 | 84 | CRP (mg/L) | Serum | Unknown | 10.16 (3.15) | 3.58 (0.97) |
| Alexandrova 2016 [8] | I | MMSE < 24 | 1 year | 20 | 11 | hsCRP (mg/L) | Serum | Discharge | 41.83 (138.61)* | 1.87 (0.96)* |
|  |  |  |  |  |  | WBC (x10^9^/L) | Serum | Admission | 8.3 (2.1) | 7.0 (1.3) |
| Wang 2021 [9] | I | MMSE < 27 | <1 week | 36 | 33 | WBC (x10^9^/L) | Serum | < 3 days | 7.29 (1.92) | 7.21 (2.00) |
| Narasimhalu 2015 [10] | I | NPA | 3-4 months | 132 | 102 | CRP (unkown) | Serum | Median 47 days | 8.44 (16.20) | 6.56 (12.41) |
|  |  |  |  |  |  | IL-1b (unkown) | Serum | Median 47 days | 0.05 (0.13)^ꭞ^ | 0.10 (0.31) |
|  |  |  |  |  |  | IL-6 (unkown) | Serum | Median 47 days | 2.50 (6.51) ^ꭞ^ | 3.69 (19.01) |
|  |  |  |  |  |  | IL-8 (unkown) | Serum | Median 47 days | 1.56 (1.64) ^ꭞ^ | 1.24 (1.34) |
|  |  |  |  |  |  | IL-10 (unknown) | Serum | Median 47 days | 2.58 (13.23) ^ꭞ^ | 1.38 (10.42) |
|  |  |  |  |  |  | IL-12 (unknown) | Serum | Median 47 days | 0.05 (0.18) ^ꭞ^ | 0.05 (0.16) |
|  |  |  |  |  |  | TNF-a (unknown) | Serum | Median 47 days | 1.66 (12.75) ^ꭞ^ | 7.31 (59.01) |
| Guoping 2015 [11] | I | MoCA < 26 | 2-3 months | 61 | 25 | IL-1b (pg/ml) | Serum | 2-3 months | 145.36 (47.90) ^ꭞ^ | 121.5 (38.8) |
|  |  |  |  |  |  | IL-2 (pg/ml) | Serum | 2-3 months | 253.30 (75.62) ^ꭞ^ | 208.6 (71.5) |
|  |  |  |  |  |  | IL-6 (pg/ml) | Serum | 2-3 months | 739.30 (162.11)^ꭞ^ | 460.8 (80.1) |
|  |  |  |  |  |  | IFN-g (pg/ml) | Serum | 2-3 months | 818.54 (107.33)^ꭞ^ | 651.2 (53.4)^ꭞ^ |
| Kulesh 2018 [12] | I | NPA | 1-2 weeks | 42 | 15 | IL-1b (ng/ml) | CSF | 4-21 days | 34.77 (111.76)^ꭞꭞ*^ | 15.34 (17.06)* |
|  |  |  |  |  |  | IL-10 (ng/ml) | CSF | 4-21 days | 19.45 (11.95)^ꭞ*^ | 23.93 (56.80)* |
|  |  |  |  |  |  | IL-6 (ng/ml) | Serum | 4-21 days | 21.00 (21.21)^ꭞ*^ | 15.00 (15.86)* |
|  |  |  |  |  |  | TNF-a (uknown) | Serum | 4-21 days | 14.94 (3.74)^ꭞ*^ | 12.62 (3.57)* |
| Zha 2022 [13] | I | MMSE < 24 | <3 months | 87 | 280 | NLR | Serum | <24 h | 2.73 (1.26) | 2.14 (0.80) |
| Bao 2023 [14] | I | MoCA < 25 | <3 months | 130 | 124 | hsCRP (mg/L) | Serum | <24 h | 6.55 (5.32)* | 5.06 (3.75)* |
| Lu 2022 [15] | I | MMSE < 26 | Immediately | 63 | 57 | IFN-g (pg/ml) | Serum | Immediately | 103.74 (58.63)* | 91.71 (47.42)* |
|  |  |  |  |  |  | IL-17A (pg/ml) | Serum | Immediately | 112.66 (64.22)* | 87.5 (32.30)* |
| Li 2022 [16] | I | MMSE < 27 | Unknown | 76 | 100 | TNF-a (pg/ml) | Serum | Unknown | 120.36 (65.49)* | 86.04 (24.81)* |
|  |  |  |  |  |  | IL-1b (pg/ml) | Serum | Unknown | 7.37 (5.66)* | 5.74 (3.71)* |
|  |  |  |  |  |  | IL-6 (pg/ml) | Serum | Unknown | 61.47 (33.50)* | 48.93 (26.88)* |
|  |  |  |  |  |  | IL-17 (pg/ml) | Serum | Unknown | 95.09 (80.86)* | 89.17 (59.94)* |
| Shang 2022 [17] | I | MoCA < 22 | 7-10 days | 253 | 201 | Lymphocytes (*10^9^) | Serum | <24 h | 1.73 (0.54)* | 1.90 (0.67)* |
|  |  |  |  |  |  | NLR | Serum | <24 h | 2.75 (1.62)* | 2.33 (1.20)* |
| Shan 2022 [18] | I | MMSE < 27 | <3 months | 137 | 139 | WBC | Serum | <24h | 7.1 (2.2) | 6.1 (1.6) |
|  |  |  |  |  |  | hsCRP | Serum | <24h | 7.11 (5.39)* | 5.80 (5.47)* |
| Zhang 2022 [19] | I | MoCA < 22 | <2 weeks | 105 | 82 | WBC | Serum | Unknown | 7.6 (2.1)* | 7.8 (2.34)* |
|  |  |  |  |  |  | Neutrophils (*10^9^) | Serum | Unknown | 5.00 (1.65)* | 4.6 (2.11)* |
|  |  |  |  |  |  | Lymphocytes (*10^9^) | Serum | Unknown | 2.07 (1.50)* | 1.90 (0.91)* |
|  |  |  |  |  |  | IL-6 (pg/ml) | Serum | Unknown | 3.89 (3.68)* | 2.65 (1.98)* |
|  |  |  |  |  |  | CRP (mg/L) | Serum | Unknown | 1.37 (1.86)* | 0.90 (1.89)* |
| Pu 2022 [20] | I | MoCA <26 | <3 months | 102 | 78 | MMP9 (ng/ml) | Serum | <24 h | 392.30 (146.10) | 280.60 (124.2) |
| Ying 2023 [21] | I | MMSE < 25 | 1 month | 77 | 74 | Neutrophils (*10^9^) | Serum | <24 h | 3.66 (1.00)* | 4.19 (1.60)* |
|  |  |  |  |  |  | Lymphocytes (*10^9^) | Serum | <24 h | 1.85 (0.59) | 2.01 (0.61) |
|  |  |  |  |  |  | hsCRP | Serum | <24 h | 2.22 (2.07)* | 2.23 (2.71)* |
|  |  |  |  |  |  | IL-10 | Serum | <24 h | 0.89 (0.72)* | 1.30 (1.13)* |
| Xu 2023 [22] | I | MMSE < 24 | <2 weeks | 382 | 210 | Lymphocytes | Serum | <24 h | 1.83 (0.61)* | 1.70 (0.56)* |

PSCI: Post-Stroke Cognitive Impaired; PSNCI: Post-Stroke Non Cognitive Impaired; MoCA: Montreal Cognitive Assessment; MMSE: Mini-Mental State Examination; SIS: Six-Item Screener; NPA: Neuropsychological Assessment; hs-CRP: high-sensitivity C-Reactive Protein; WBC: White Blood Count; IL: Interleukin; TNF-a: Tumor Necrosis Factor Alpha; IFN-g: Interferon gamma; NLR: Neutrophil-Lymphocyte Ratio; MMP-9: Matrix Metalloproteinase 9
* Means and SD calculated from Median and IQR
^ꭞ^ Combined means and SD of multiple cohorts

**Supplementary table 2. Characteristics of included human studies on correlations between cognition and inflammation after stroke**

| **Author** | **Stroke** | **Population (n)** | **Evaluation Cognitive Impairment** | **Timing cognitive evaluation post-stroke** | **Inflammatory biomarker (concentration)** | **Biomarker obtained from** | **Timing Inflammatory biomarker post-stroke** | **Correlation** | **Remarks** |
| --- | --- | --- | --- | --- | --- | --- | --- | --- | --- |
| Nguyen 2020 [23] | I | 156 | MoCA | 3 months | WBC (x10^9^ cells/L) | Serum | 0-12h | -0.03 | Covaried for age & NIHSS |
|  |  |  |  |  |  |  | 24-48h | 0.26 | Covaried for age & NIHSS |
|  |  |  |  |  |  |  | 3-7d | -0.15 | Covaried for age & NIHSS |
|  |  | 156 | MoCA | 3 months | NC (x10^9^ cells/L) | Serum | 0-12h | 0.02 | Covaried for age & NIHSS |
|  |  |  |  |  |  |  | 24-48h | 0.29 | Covaried for age & NIHSS |
|  |  |  |  |  |  |  | 3-7d | -0.18 | Covaried for age & NIHSS |
| Kliper 2013 [24] | I | 255 | NPA | <4 days | CRP (unknown) | Serum | <3 days, 6 & 12 months | -0.283 | LNCRP vs total cognition LNCRP vs MoCA was not associated |
|  |  |  | NPA | <4 days | WBC (unknown) | Serum | <3 days, 6 & 12 months | -0.180 | WBC vs total cognition WBC vs MoCA was not associated |
| Guoping 2015 [11] | I | 61 | VDAS-Cog | 2-3 months | IL-6 (ng/ml) | Serum | 2-3 months | -0.63 |  |
| Kulesh 2018 [12] | I | 57 | MMSE | 2-3 weeks | IL-1b (ng/ml) | Serum | 4-21 days | -0.45^∆^ | Other correlations:  No data shown |
|  |  |  |  |  | IL-10 (ng/ml) | Serum | 4-21 days | -0.19^∆^ |  |
| Spaletta 2013 [25] | I | 48 | MMSE | 3 days | IL-6 (pg/ml) | Serum | 3 days | -0.066 |  |
| Shaheen 2019 [26] | I | 44 | MMSE | <2 days | IL-8 (unknown) | Serum | <2 days | -0.27 |  |
|  |  |  | MoCA | <2 days | IL-8 (unknown) | Serum | <2 days | -0.43 |  |
| Rothenburg 2010 [27] | I | 48 | MMSE | 5-30 days | CRP (ng/ml) | Serum | 5-30 days | -0.46 |  |
|  |  |  |  |  | IL-6 (pg/ml) | Serum | 5-30 days | -0.33 |  |
|  |  |  |  |  | IFN-g (pg/ml) | Serum | 5-30 days | -0.047 |  |
| Lu 2022 [15] | I | 120 | MMSE | Immediately | IFN-g (pg/ml) | Serum | Immediately | -0.154 |  |
|  |  |  |  |  | IL-17A (pg/ml) | Serum | Immediately | -0.267 |  |
| Li 2022 [16] | I | 176 | MMSE | <1 month | TNF-a | Serum | <1 month | -0.33 |  |
|  |  |  |  |  | IL-1b | Serum | <1 month | -0.137 |  |
|  |  |  |  |  | IL-6 | Serum | <1 month | -0.227 |  |
|  |  |  |  |  | IL-17 | Serum | <1 month | -0.067 |  |
| Pu 2022 [20] | I | 180 | MoCA | <3 months | MMP9 (pg/ml) | Serum | <24 h | -0.54 |  |
| Ying 2023 [21] | I | 151 | MMSE | 1 month | IL-10 (pg/ml) | Serum | <24 h | -0.169 |  |
| Wang 2022 [28] | I | 120 | MMSE | Unknown | IL-17A | Serum | <24 h | -0.442 |  |

I: Ischemic; MoCA: Montreal Cognitive Assessment; NPA: Neuropsychological Assessment; VDAS-Cog: Vascular Dementia Assessment Scale-cognition; MMSE: Mini-Mental State Examination; WBC: White Blood Cells; NC: Neutrophil Count; CRP: C-reactive protein; IL: Interleukin; IFN-g: Interferon Gamma; TNF-a: Tumor Necrosis Factor Alpha; MMP9: Matrix Metalloproteinase 9; NIHSS: National Institute of Health Stroke Scale
^∆^ Calculated from Figure

**Supplementary table 3. Characteristics of included animal studies**

| **Author** | **Stroke** | **Strain & species** | **Animal Age (weeks)** | **Animal Sex** | **Subgroup** | **Intervention** | **Timing First Intervention** | **Cognitive Test** | **Timing Cognitive assessment (days)** |
| --- | --- | --- | --- | --- | --- | --- | --- | --- | --- |
| Liao 2020 [29] | I | Sprague Dawley Rats | Unknown | Male | General Anti-inflammatory | Tanshinol Borneol Ester (DBZ) | 1 hour pre-stroke | MWM EL day 3 MWM EL final day MWM TQT RAWM errors | 6 -8 |
| Kim 2020 [30] | I | C57BL/6 Mice | 6-72 | Male | General Anti-inflammatory | Absent in Melanoma 2 (AIM2) knockout | Transgenic | MWM EL day 3 MWM EL final day MWM TQT | 24 -28 |
| Qu 2018 [31] | I | Wistar Rats | Unknown | Male | General Anti-inflammatory | Arachidonic Acid | 24 hours post-stroke | MWM EL day 3 MWM EL final day | 3-6 |
| Xu 2019 [32] | I | Sprague Dawley Rats | Unknown | Male | General Anti-inflammatory | Aspirin | Immediately after stroke | PAT Errors  MWM EL day 3  MWM TQT | 14 |
| Chin 2013 [33] | I | C57BL/6 Mice | Adult | Male | General Anti-inflammatory | Purinergic Receptor P2Y1 knockout | Transgenic | CoFC % Freezing | 4-7 |
| Chin 2013 (2) [33] | I | C57BL/6 Mice | Adult | Male | General Anti-inflammatory | MRS2500 (P2Y1 antagonist) | Immediately after stroke | CoFC % Freezing | 4 |
| Guo 2016 [34] | SAH | Sprague Dawley Rats | Unknown | Male | General Anti-inflammatory | Lipoxin A4 (LXA4) | 1.5 hours post-stroke | MWM EL day 3  MWM EL final day  MWM TQT T-Maze alterations | 24-26 |
| Xu 2020 [35] | SAH | Sprague Dawley Rats | Unknown | Unknown | General Anti-inflammatory | BMS-470539 (MC1 receptor agonist) | 1 hour after stroke | MWM EL day 3  MWM EL final day  MWM TQT | 23-25 |
| Li 2020 (2) [36] | I | C57BL/6 Mice | 56 | Unknown | General Anti-inflammatory | MCC950 (NLRP3 inhibitor) | 0.5 hours post-stroke | MWM EL day 3  MWM EL final day  MWM TQT | 32-35 |
| Yang 2015 [37] | ICH | Sprague Dawley Rats | Unknown | Male | General Anti-inflammatory | Indometacin | 2 days pre-stroke | MWM EL day 3  MWM TQT | 3-5 |
| Balkaya 2021 [38] | I | C57BL/6 Mice | 12-16 | Male, Female | General Anti-inflammatory | Cluster of differentiation 36 (CD36)-knockout | Transgenic | MWM EL day 3 MWM EL final day MWM TQT | 3-7 |
| Guo 2021 [39] | I | Sprague Dawley Rats | Adult | Unknown | General anti-inflammatory | Celecoxib | 24h post-stroke | MWM EL day 3 MWM EL final day MWM TQT | 7-10 |
| Fang 2022 [40] | SAH | Sprague Dawley Rats | Adult | Male | General anti-inflammatory | VX-765 (Caspase-1 inhibitor) | 1h post-stroke | MWM EL day 3 MWM EL final day MWM TQT | 25-28 |
| Cao 2023 [41] | SAH | Sprague-Dawley Rats | 8-10 | Male | General anti-inflammatory | MCC950 (NLRP3 inhibitor) | 1h post-stroke | MWM EL final day MWM TQT Y-maze alterations NOR time at novel | 14-20 |
| Qin 2023 [42] | I | C57BL/6J mice | 7-8 | Male | General anti-inflammatory | Tie1-MKP-1 (MKP-1 overexpression) | 5 days pre-stroke | NOR discrimination | 14 |
| Ortega 2020 [43] | I | C57BL/6 Mice | 8-16 | Male | B cell depletion | Rituximab | 3 days pre-stroke | NOR Time at novel  NOR preference CoFC % Freezing CuFM % Freezing | 70 |
| Doyle 2015 [44] | I | C57BL/6 Mice | 12-20 | Male | B cell depletion | MuMT (B-cell deficient) | Transgenic | OLT % rears moved  Y-maze alterations | 7-49 |
| Doyle 2015 (2) [44] | I | C57BL/6 Mice | 12-20 | Male | B cell depletion | Anti-CD20 (B-lymphocyte antigen) | 5 days post-stroke | OLT % rears moved | 7-49 |
| Shi 2019 [45] | ICH | C57BL/6 Mice | 12-16 | Male | Microglia depletion | PLX3397 (inhibitor of colony stimulating factor 1 receptor (CSF1R)) | 7 days post-stroke | MWM EL day 3  MWM EL final day MWM TQT | 38-40 |
| Costa 2021 [46] | I | C57BL/6 Mice | 12-14 | Male, Female | Microglia depletion | M3RKOmi (deletion of muscarinic acetylcholine receptor 3 in microglia) | Transgenic | BM TTB day 3  BM TTB final day  NOR Time at novel | 22-27 |
| Jia 2023 [47] | I | C57BL/6 mice | Unknown | Unknown | Microglia depletion | rAAV-taCasp3 (partial depletion of CD11c+ microglia) | 3 weeks pre-stroke | MWM EL day 3 MWM EL final day MWM TQT | 18-21 |
| Islam 2022 [48] | SAH | C57BL/6 mice | 8-12 | Male | Microglia depletion | Clodronate | 1-2 days post-stroke | BM TTB day 3 | 6 |
| Ge 2017 [49] | I | C57BL/6 Mice | Adult | Male | Microglia phenotype | MDM (monocyte-derived macrophages) | 1 day post-stroke | AAT avoidances | 20-96 |
| Wang 2020 (2) [50] | I | C57BL/6 Mice | 10-12 | Male | Microglia phenotype | TAK1 mKO (microglia/macrophage-specific knockout of transforming growth factor-β-activated kinase 1 (TAK1)) | Transgenic | MWM EL day 3  MWM EL final day  MWM TQT | 24-27 |
| Wang 2020 (3) [50] | I | C57BL/6 Mice | 10-12 | Male | Microglia phenotype | 5Z-7-OZ (5Z-7-Oxozeaenol) | 2 hours post-stroke | MWM EL day 3  MWM EL final day  MWM TQT | 24-27 |
| Xu 2021 (2) [51] | SAH | C57BL/6 Mice | Unknown | Male | Microglia phenotype | LP17 (inhibitor of triggering receptor expressed on myeloid cells 1 (TREM-1)) | 1 hour post-stroke | MWM EL day 3  MWM EL final day  MWM TQT | 25-28 |
| Xu 2021 (3) [51] | SAH | C57BL/6 mice | Uknown | Male | Microglia phenotype | 5Z-7-OZ (5Z-7-Oxozeaenol) | 30 mins post-stroke | MWM EL day 3 MWM EL final day MWM TQT | 25-28 |
| Rynkowski 2009 [52] | ICH | C57BL/6 Mice | Unknown | Male | Complement Inhibition | C3aRA (C3a-receptor antagonist) | 45 mins pre-stroke | MWM TQT | 3 |
| Rynkowski 2009 (2) [52] | ICH | C57BL/6 Mice | Unknown | Male | Complement Inhibition | C3aRA (C3a-receptor antagonist) | 6 hours post-stroke | MWM TQT | 3 |
| Alawieh 2018 [53] | I | C57BL/6 Mice | 56 | Male, Female | Complement Inhibition | B4Crry (complement inhibitor) | 2 hours post-stroke | BM LTB day 3  BM LTB final day  PAT EL day 3  PAT EL final day | 3-15 |
| Alawieh 2018 (2) [53] | I | C57BL/6 Mice | 56 | Male, Female | Complement Inhibition | B4Crry (complement inhibitor) | 6 hours post-stroke | BM LTB day 3  BM LTB final day  PAT EL day 3  PAT EL final day | 3-15 |
| Alawieh 2018 (3) [53] | I | C57BL/6 Mice | 56 | Male, Female | Complement Inhibition | B4Crry (complement inhibitor) | 24 hours post-stroke | BM LTB day 3  BM LTB final day | 12-15 |
| Alawieh 2020 [54] | I | C57BL/6 Mice | 12 | Male | Complement Inhibition | B4Crry (complement inhibitor) | Immediately after stroke | BM LTB day 3  BM LTB final day  BM errors day 3  BM errors final day | 26-30 |
| Garrett 2009 [55] | ICH | C57BL/6 Mice | Unknown | Male | Complement Inhibition | C5aRA (C5a receptor antagonist) | 6 hours post-stroke | MWM TQT | 3 |
| Garrett 2009 (2) [55] | ICH | C57BL/6 Mice | Unknown | Male | Complement Inhibition | C3aRA + C5aRA (C3a + C5a receptor antagonist) | 6 hours post-stroke | MWM TQT | 3 |
| Shi 2019 (2) [45] | ICH | C57BL/6 Mice | 12-16 | Male | Fingolimod | FTY720 (Fingolimod) | 7 days post-stroke | MWM EL day 3  MWM EL final day  MWM TQT | 38-40 |
| Wang 2020 [56] | SAH | C57BL/6 Mice | Unknown | Male | Fingolimod | FTY720 (Fingolimod) | 2 hours post-stroke | MWM EL day 3  MWM EL final day  MWM TQT | 16-19 |
| Rolland 2013 [57] | ICH | Sprague Dawley Rats | Unknown | Male | Fingolimod | FTY720 (Fingolimod) | 1 hour post-stroke | MWM distance  MWM to target  MWM from target | 58-60 |
| Yang 2019 [58] | ICH | ICR Mice | Adult | Male | Fingolimod | FTY720 (Fingolimod) | 0.5 hours post-stroke | NOR discrimination | 28 |
| Zhang 2022 [19] | I | Sprague-Dawley Rats | Unknown | Male | Fingolimod | FTY720 | 7 days pre-stroke | Y-maze correct | Unknown |
| Chu 2010 [59] | I | Sprague Dawley Rats | Unknown | Male | Minocycline | Minocycline | 2 hours post-stroke | RAWM errors day 3  RAWM errors final | 32-35 |
| Li 2021 [60] | I | Sprague Dawley Rats | Adult | Male | Minocycline | Minocycline | 6 hours post-stroke | MWM TQT | 28 |
| Li 2021 (2) [60] | I | Sprague Dawley Rats | Adult | Male | Minocycline | Minocycline | 24 hours post-stroke | MWM TQT | 28 |
| Miao 2018 [61] | ICH | Sprague Dawley Rats | Unknown | Unknown | Minocycline | Minocycline | 15 min post-stroke | MWM TQT | 32 |
| Hosseini 2018 [62] | I | Wistar Rats | Unknown | Male | LPS | LPS (liposaccharide) | 2 days pre-stroke | RAWM EL day 3  RAWM EL final day  RAWM Errors RAWM Goal Time | 3-4 |
| Hosseini 2018 (2) [62] | I | Wistar Rats | Unknown | Male | LPS | MPL (monophosphoryl lipid A) | 2 days pre-stroke | RAWM EL day 3  RAWM EL final day  RAWM Errors RAWM Goal Time | 3-4 |
| Li 2020 [36] | I | C57BL/6 Mice | 56 | Unknown | LPS | LPS (liposaccharide) | 1 day pre-stroke | MWM EL day 3  MWM EL final day  MWM TQT | 32-25 |
| Xu 2020 (2) [63] | ICH | Sprague Dawley Rats | Unknown | Unknown | IL-4 | IL-4 (interleukin 4) | 2 hours post-stroke | MWM EL day 3  MWM EL final day  MWM TQT | 18-21 |
| Liu 2016 [64] | I | C57BL/6 Mice | Unknown | Male | IL-4 | IL-4 (interleukin 4) knockout | Transgenic | MWM EL day 3  MWM EL final day  MWM TQT | 19-21 |
| Liu 2016 (2) [64] | I | C57BL/6 Mice | Unknown | Male | IL-4 | IL-4 (interleukin 4) | 6 hours post-stroke | MWM EL day 3  MWM EL final day  MWM TQT | 25-28 |
| Zhang 2019 [65] | I | C57BL/6 Mice | 8 | Male | IL-4 | IL-4 (interleukin 4) | 6 hours post-stroke | MWM EL day 3  MWM EL final day  MWM TQT | 26-28 |
| Wu 2018 [66] | I | Sprague Dawley Rats | Adult | Male | Other | rGAS6 (recombinant growth arrest-specific 6) | 1 hour post-stroke | MWM EL day 3  MWM EL final day  MWM TQT | 26-28 |
| Ma 2016 [67] | ICH | ICR Mice | Adult | Male | Other | Anti-B7-1 (monoclonal anti-B7 antibody) | 10 min post-stroke | MWM EL day 3  MWM EL final day | 10-21 |
| Jianrong 2019 [68] | I | C57BL/6 Mice | Unknown | Male | Other | LV-DUSP (lentiviral dual-specificity phosphatase) | 14 days pre-stroke | MWM crossovers  MWM EL day 3  MWM EL final day  MWM TQT | 25-28 |
| Wei 2017 [69] | I | C57BL/6 Mice | Unknown | Male | Other | MR16-1 (anti-interleukin-6 receptor antibody) | Immediately post-stroke | BM TTB  MWM TQT  Y-maze correct | 26 |
| Xie 2021 [70] | I | C57BL/6 Mice | Young | Male | Other | ST2 (interleukin 1 receptor-like 1) knockout | Transgenic | NOR discrimination  NOR recognition  MWM EL day 3  MWM EL final day  MWM TQT | 10-27 |
| Xu 2020 (3) [63] | ICH | Sprague Dawley Rats | Unknown | Unknown | Other | STAT6 (signal transducer and activator of transcription 6) knockout | Transgenic | MWM EL day 3  MWM EL final day  MWM TQT | 18-21 |
| Pettigrew 2016 [71] | I | Sprague Dawley Rats | Unknown | Male | Other | TNF-α (tumor necrosis factor α) overexpression | Transgenic | MWM EL day 3  MWM EL final day  MWM TQT | 7 |
| Xiong 2016 [72] | ICH | C57BL/6 Mice | Unknown | Male | Other | TAK242 (selective Toll-like receptor 4 inhibitor) | 6 hours post-stroke | RAWM errors day 3  RAWM errors final | 8-10 |
| Stagliano 1997 [73] | I | Wistar Rats | Unknown | Male | Other | L-NAME (L-N^G^-Nitro arginine ester) | Immediately post-stroke | MWM EL final day  MWM TQT | 2-21 |
| Stagliano 1997 (2) [73] | I | Wistar Rats | Unknown | Male | Other | SIN-1 (peroxynitrite donor) | Immediately post-stroke | MWM TQT | 2 |
| Li 2018 [1] | I | C57BL/6 Mice | 10-14 | Male | Other | eNOS (endothelial nitric oxide synthase) knockout | Transgenic | MWM EL day 3  MWM EL final day  MWM TQT | 24-27 |
| Chen 2022 [74] | I | C57BL/6 mice | 8-10 | Unknown | Other | IL-13 | 2h post-stroke | MWM EL day 3 MWM EL final day MWM TQT | 32-35 |
| Li 2023 [75] | I | C57BL/6 mice | Unknown | Male | Other | LCN-2 knockout | Trangenic | MWM EL day 3 MWM EL final day MWM TQT MWM crossovers | 25-28 |

I: Ischemic Stroke; ICH: Intracerebral Heamorrhage; SAH: Subarachnoid Haemorrhage; MWM: Morris Water Maze; RAWM: Radial Arm Water Maze; NOR: Novel Object Recognition; CoFC: Contextual Fear Conditioning; CuFM: Conditional Fear Memory; BM: Barnes Maze; AAT: Active Avoidance Task; PAT: Passive Avoidance Task; EL: escape latency; TQT: Target Quadrant Time; LTB: Length to Box

**Supplementary table 4. Quality assessment of included human studies**

| **Author** | **Selection** | | | | **Comparability** | **Outcome** | | |
| --- | --- | --- | --- | --- | --- | --- | --- | --- |
|  | Adequate case description | Representative series of cases | Selection of controls | Definition of controls | Comparability of cohorts | Assessment of inflammation | Same assessment in controls | Non-response rate |
| Li 2018 | * |  | * | * |  | * | * |  |
| Nguyen 2020 | * |  | * | * | ** | * | * |  |
| Zhu 2020 | * |  | * | * | ** | * | * |  |
| Guoping 2015 | * |  | * | * | ** | * | * |  |
| Kliper 2013 |  | * |  |  | ** | * | * |  |
| Alexandrova 2016 | * |  | * | * | ** | * | * |  |
| Kulesh 2018 |  |  |  | * | ** | * | * |  |
| Guo 2018 |  | * | * | * |  | * | * |  |
| Liu 2019 | * |  |  | * | ** | * | * |  |
| Narasimhalu 2015 | * | * | * | * | ** | * | * |  |
| Spaletta 2013 | * | * | * |  | * | * | * |  |
| Shaheen 2019 | * |  |  |  | ** | * | * |  |
| Wang 2021 | * |  | * | * | ** | * | * |  |
| Mao 2020 | * |  | * |  | ** | * | * |  |
| Yang 2021 |  |  | * | * | ** | * | * |  |
| Ran 2020 |  |  | * | * |  | * | * |  |
| Rothenburg 2010 | * |  | * | * |  | * | * |  |
| Zha 2022 | * | * | * | * | * | * | * |  |
| Bao 2023 | * |  | * | * | * | * | * |  |
| Lu 2022 | * | * | * | * |  | * | * |  |
| Shang 2022 | * |  | * | * | * | * | * |  |
| Shan 2022 | * |  | * | * |  | * | * |  |
| Pu 2022 | * |  | * | * | ** | * | * |  |
| Ying 2023 | * |  | * | * | ** | * | * |  |
| Xu 2023 | * | * | * | * |  | * | * |  |
| Li 2022 | * |  | * | * |  | * | * |  |
| Wang 2022 | * |  | * | * | ** | * | * |  |
| Zhang 2022 | * |  | * | * | ** | * | * |  |

**Supplementary table 4. Risk of bias of included animal studies**

| **Author** | **Selection** | | | **Performance** | | **Detection** | | **Attrition** | **Reporting** |
| --- | --- | --- | --- | --- | --- | --- | --- | --- | --- |
|  | Random group allocation | Groups similar at baseline | Blinded group allocation | Random housing | Blinded interventions | Random outcome assessment | Blinded outcome assessment | Reporting of drop-outs | Selective outcome reporting |
| Liao 2020 | - | ? | ? | ? | - | ? | - | ? | ? |
| Xu 2020 | - | ? | ? | ? | - | ? | ? | ? | ? |
| Kim 2020 | - | ? | - | ? | - | ? | - | ? | ? |
| Qu 2018 | - | ? | - | ? | ? | ? | ? | ? | ? |
| Wu 2018 | - | ? | ? | ? | - | ? | - | ? | ? |
| Ortega 2020 | - | ? | ? | ? | - | ? | - | ? | ? |
| Doyle 2015 | - | ? | ? | ? | ? | ? | - | ? | ? |
| Ma 2016 | - | ? | - | ? | ? | ? | ? | ? | ? |
| Rynkowski 2009 | ? | ? | - | ? | ? | ? | - | ? | ? |
| Balkaya 2021 | - | ? | - | ? | - | ? | ? | ? | ? |
| Ge 2017 | - | ? | - | ? | ? | - | - | ? | ? |
| Shi 2019 | - | ? | - | ? | - | ? | - | ? | ? |
| Xu 2019 | - | ? | ? | ? | ? | ? | ? | ? | ? |
| Alawieh 2020 | - | ? | - | ? | - | ? | - | ? | ? |
| Costa 2021 | ? | ? | - | ? | ? | ? | - | ? | ? |
| Jianrong 2019 | ? | ? | ? | ? | ? | ? | ? | ? | ? |
| Wei 2017 | - | ? | - | ? | - | ? | - | ? | ? |
| Wang 2020 | ? | ? | ? | ? | - | ? | ? | ? | ? |
| Rolland 2013 | - | ? | - | ? | - | ? | - | ? | ? |
| Yang 2019 | ? | ? | ? | ? | - | ? | - | ? | ? |
| Xu 2020 (2) | - | ? | ? | ? | - | ? | ? | ? | ? |
| Xie 2021 | - | ? | - | ? | - | ? | - | ? | ? |
| Li 2018 | ? | ? | - | ? | - | ? | - | ? | ? |
| Liu 2016 | - | ? | - | ? | ? | ? | ? | ? | ? |
| Chin 2013 | ? | ? | ? | ? | ? | ? | ? | ? | ? |
| Guo 2016 | ? | ? | ? | ? | ? | ? | - | ? | ? |
| Li 2021 | ? | ? | ? | ? | ? | ? | ? | ? | ? |
| Chu 2010 | ? | ? | ? | ? | ? | ? | ? | ? | ? |
| Miao 2018 | - | ? | - | ? | ? | ? | ? | ? | ? |
| Hosseini 2018 | - | ? | ? | ? | ? | ? | ? | ? | ? |
| Garrett 2009 | - | ? | - | ? | - | - | - | ? | ? |
| Alawieh 2018 | - | ? | - | - | - | ? | - | ? | ? |
| Li 2020 | - | ? | ? | ? | - | ? | ? | ? | ? |
| Zhang 2019 | - | ? | - | ? | - | ? | - | ? | ? |
| Stagliano 1997 | - | ? | ? | - | ? | ? | - | ? | ? |
| Pettigrew 2016 | ? | ? | ? | ? | - | ? | - | ? | ? |
| Yang 2015 | ? | ? | ? | ? | - | ? | - | ? | ? |
| Xiong 2016 | ? | - | ? | ? | ? | ? | ? | + | ? |
| Wang 2020 (2) | - | ? | - | ? | - | ? | - | ? | ? |
| Xu 2021 | - | ? | ? | ? | - | ? | ? | ? | ? |
| Xu 2021 (3) | - | - | - | ? | - | ? | - | - | ? |
| Jia 2023 | ? | ? | ? | ? | ? | ? | - | ? | ? |
| Chen 2022 | - | ? | - | ? | - | ? | - | ? | ? |
| Fang 2022 | - | ? | - | ? | - | ? | - | - | ? |
| Guo 2021 | ? | ? | ? | ? | ? | ? | ? | ? | ? |
| Islam 2022 | ? | ? | ? | ? | - | ? | - | ? | ? |
| Zhang 2022 | - | ? | ? | ? | - | ? | - | ? | ? |
| Qin 2023 | - | ? | - | ? | - | ? | - | ? | ? |
| Cao 2023 | - | ? | - | ? | ? | ? | ? | ? | ? |
| Li 2023 | - | ? | - | ? | - | ? | ? | ? | ? |

- : Low risk of bias
 ? : Unclear risk of bias
 + : High risk of bias

**References**

1. Li S, Wang Y, Jiang Z, Huai Y, Liao JK, Lynch KA, et al. Impaired Cognitive Performance in Endothelial Nitric Oxide Synthase Knockout Mice After Ischemic Stroke: A Pilot Study. American Journal of Physical Medicine & Rehabilitation. 2018;97:492–9.

2. Zhu C, Li G, Lv Z, Li J, Wang X, Kang J, et al. Association of plasma trimethylamine-N-oxide levels with post-stroke cognitive impairment: a 1-year longitudinal study. Neurol Sci. 2020;41:57–63.

3. Mao L, Chen X-H, Zhuang J-H, Li P, Xu Y-X, Zhao Y-C, et al. Relationship between β-amyloid protein 1-42, thyroid hormone levels and the risk of cognitive impairment after ischemic stroke. WJCC. 2020;8:76–87.

4. Ran F, Liu F, Zhang Y, Chen L. Serum Uric Acid and High-Sensitivity C-Reactive Protein as Predictors of Cognitive Impairment in Patients with Cerebral Infarction. Dement Geriatr Cogn Disord. 2020;49:235–42.

5. Liu Y, Chen H, Zhao K, He W, Lin S, He J. High levels of plasma fibrinogen are related to post-stroke cognitive impairment. Brain Behav. 2019;9:e01391.

6. Guo J, Su W, Fang J, Chen N, Zhou M, Zhang Y, et al. Elevated CRP at admission predicts post-stroke cognitive impairment in Han Chinese patients with intracranial arterial stenosis. Neurological Research. 2018;40:292–6.

7. Yang G, Li C, Wang W, Wang C, Dong A, Wang F, et al. Risk factors for cognitive impairment in patients with first-time ischemic stroke. Am J Transl Res. 2021;13:1884–9.

8. Alexandrova ML, Danovska MP. Cognitive impairment one year after ischemic stroke: predictorsand dynamics of significant determinants. Turk J Med Sci. 2016;46:1366–73.

9. Wang X, Miao Z, Xu X, Schultzberg M, Zhao Y. Reduced Levels of Plasma Lipoxin A4 Are Associated with Post-Stroke Cognitive Impairment. JAD. 2021;79:607–13.

10. Narasimhalu K, Lee J, Leong Y-L, Ma L, De Silva DA, Wong M-C, et al. Inflammatory Markers and Their Association with Post Stroke Cognitive Decline. International Journal of Stroke. 2015;10:513–8.

11. Guoping P, Wei W, Xiaoyan L, Fangping H, Zhongqin C, Benyan L. Characteristics of the peripheral T cell immune response of patients at different stages of vascular cognitive impairment. Immunology Letters. 2015;168:120–5.

12. Kulesh A, Drobakha V, Kuklina E, Nekrasova I, Shestakov V. Cytokine Response, Tract-Specific Fractional Anisotropy, and Brain Morphometry in Post-Stroke Cognitive Impairment. Journal of Stroke and Cerebrovascular Diseases. 2018;27:1752–9.

13. Zha F, Zhao J, Chen C, Ji X, Li M, Wu Y, et al. A High Neutrophil-to-Lymphocyte Ratio Predicts Higher Risk of Poststroke Cognitive Impairment: Development and Validation of a Clinical Prediction Model. Front Neurol. 2022;12:755011.

14. Bao Y, Wang L, Du C, Ji Y, Dai Y, Jiang W. Association between Systemic Immune Inflammation Index and Cognitive Impairment after Acute Ischemic Stroke. Brain Sciences. 2023;13:464.

15. Lu T, Ma L, Xu Q, Wang X. Blood Th17 cells and IL‐17A as candidate biomarkers estimating the progression of cognitive impairment in stroke patients. Clinical Laboratory Analysis. 2022;36:e24581.

16. Li R, Fan W, Li D, Liu X. Correlation of common inflammatory cytokines with cognition impairment, anxiety, and depression in acute ischemic stroke patients. Braz J Med Biol Res. 2022;55:e11517.

17. Shang T, Ma B, Shen Y, Wei C, Wang Z, Zhai W, et al. High neutrophil percentage and neutrophil-lymphocyte ratio in acute phase of ischemic stroke predict cognitive impairment: A single-center retrospective study in China. Front Neurol. 2022;13:907486.

18. Shan W, Xu L, Xu Y, Qiu Z, Feng J, Zhao J, et al. Leukoaraiosis Mediates the Association of Total White Blood Cell Count With Post-Stroke Cognitive Impairment. Front Neurol. 2022;12:793435.

19. Zhang M-S, Liang J-H, Yang M-J, Ren Y-R, Cheng D-H, Wu Q-H, et al. Low Serum Superoxide Dismutase Is Associated With a High Risk of Cognitive Impairment After Mild Acute Ischemic Stroke. Front Aging Neurosci. 2022;14:834114.

20. Pu M, You Y, Wang X. Predictive value of serum matrix metalloproteinase 9 combined with tissue inhibitor of metalloproteinase 1 for post-stroke cognitive impairment. Journal of Clinical Neuroscience. 2022;105:103–8.

21. Ying Z, Huang Y-Y, Shao M-M, Chi C-H, Jiang M-X, Chen Y-H, et al. Relationships of Low Serum Levels of Interleukin-10 With Poststroke Anxiety and Cognitive Impairment in Patients With Clinical Acute Stroke. J Clin Neurol. 2023;19:242.

22. Xu M, Chen L, Hu Y, Wu J, Wu Z, Yang S, et al. The HALP (hemoglobin, albumin, lymphocyte, and platelet) score is associated with early-onset post-stroke cognitive impairment. Neurol Sci. 2023;44:237–45.

23. Nguyen VA, Crewther SG, Howells DW, Wijeratne T, Ma H, Hankey GJ, et al. Acute Routine Leukocyte and Neutrophil Counts Are Predictive of Poststroke Recovery at 3 and 12 Months Poststroke: An Exploratory Study. Neurorehabil Neural Repair. 2020;34:844–55.

24. Kliper E, Bashat DB, Bornstein NM, Shenhar-Tsarfaty S, Hallevi H, Auriel E, et al. Cognitive Decline After Stroke: Relation to Inflammatory Biomarkers and Hippocampal Volume. Stroke. 2013;44:1433–5.

25. Spalletta G, Cravello L, Imperiale F, Salani F, Bossù P, Picchetto L, et al. Neuropsychiatric Symptoms and Interleukin-6 Serum Levels in Acute Stroke. JNP. 2013;25:255–63.

26. Shaheen HA, Daker LI, Abbass MM, Abd El Fattah AA. Post-stroke executive dysfunction and verbal fluency negatively correlated to IL8. Egypt J Neurol Psychiatry Neurosurg. 2019;55:45.

27. Rothenburg LS, Herrmann N, Swardfager W, Black SE, Tennen G, Kiss A, et al. The Relationship Between Inflammatory Markers and Post Stroke Cognitive Impairment. J Geriatr Psychiatry Neurol. 2010;23:199–205.

28. Wang C, Huo H, Li J, Zhang W, Liu C, Jin B, et al. The longitudinal changes of serum JKAP and IL‐17A , and their linkage with anxiety, depression, and cognitive impairment in acute ischemic stroke patients. Clinical Laboratory Analysis. 2022;36:e24762.

29. Liao S, Wu J, Liu R, Wang S, Luo J, Yang Y, et al. A novel compound DBZ ameliorates neuroinflammation in LPS-stimulated microglia and ischemic stroke rats: Role of Akt(Ser473)/GSK3β(Ser9)-mediated Nrf2 activation. Redox Biology. 2020;36:101644.

30. Kim H, Seo JS, Lee S-Y, Ha K-T, Choi BT, Shin Y-I, et al. AIM2 inflammasome contributes to brain injury and chronic post-stroke cognitive impairment in mice. Brain, Behavior, and Immunity. 2020;87:765–76.

31. Qu Y, Zhang H-L, Zhang X-P, Jiang H-L. Arachidonic acid attenuates brain damage in a rat model of ischemia/reperfusion by inhibiting inflammatory response and oxidative stress. Hum Exp Toxicol. 2018;37:135–41.

32. Xu D, Xia N, Hou K, Li F, Chen S, Hu Y, et al. Clematichinenoside Facilitates Recovery of Neurological and Motor Function in Rats after Cerebral Ischemic Injury through Inhibiting Notch/NF-κB Pathway. Journal of Stroke and Cerebrovascular Diseases. 2019;28:104288.

33. Chin Y, Kishi M, Sekino M, Nakajo F, Abe Y, Terazono Y, et al. Involvement of glial P2Y1 receptors in cognitive deficit after focal cerebral stroke in a rodent model. Journal of Neuroinflammation. 2013;10:860.

34. Guo Z, Hu Q, Xu L, Guo Z-N, Ou Y, He Y, et al. Lipoxin A4 Reduces Inflammation Through Formyl Peptide Receptor 2/p38 MAPK Signaling Pathway in Subarachnoid Hemorrhage Rats. Stroke. 2016;47:490–7.

35. Xu W, Mo J, Ocak U, Travis ZD, Enkhjargal B, Zhang T, et al. Activation of Melanocortin 1 Receptor Attenuates Early Brain Injury in a Rat Model of Subarachnoid Hemorrhage viathe Suppression of Neuroinflammation through AMPK/TBK1/NF-κB Pathway in Rats. Neurotherapeutics. 2020;17:294–308.

36. Li Y-Q, Chen J-X, Li Q-W, Xiao Z-J, Yuan T, Xie Z-H. Targeting NLRP3 inflammasome improved the neurogenesis and post-stroke cognition in a mouse model of photothrombotic stroke. NeuroReport. 2020;31:806–13.

37. Yang Y, Zhang M, Kang X, Jiang C, Zhang H, Wang P, et al. Thrombin-induced microglial activation impairs hippocampal neurogenesis and spatial memory ability in mice. Behav Brain Funct 2015;11:1774

38. Balkaya M, Kim I, Shakil F, Cho S. CD36 deficiency reduces chronic BBB dysfunction and scar formation and improves activity, hedonic and memory deficits in ischemic stroke. J Cereb Blood Flow Metab. 2021;41:486–501.

39. Gou J, Liang S, Cheng W, Wu S, Ye Z, Ma Y, et al. Neuroprotective effect of combined use of nicotine and celecoxib by inhibiting neuroinflammation in ischemic rats. Brain Research Bulletin. 2021;175:234–43.

40. Fang Y, Wang X, Lu J, Shi H, Huang L, Shao A, et al. Inhibition of caspase-1-mediated inflammasome activation reduced blood coagulation in cerebrospinal fluid after subarachnoid haemorrhage. eBioMedicine. 2022;76:103843.

41. Cao Y, Wang Y, Li X, Yang X, Zeng B, Guo Z. MCC950 ameliorates cognitive function by reducing white matter microstructure damage in rats after SAH. Brain Research Bulletin. 2023;202:110743.

42. Qin X-D, Yang T-Q, Zeng J-H, Cai H-B, Qi S-H, Jiang J-J, et al. Overexpression of mitogen-activated protein kinase phosphatase-1 in endothelial cells reduces blood-brain barrier injury in a mouse model of ischemic stroke. Neural Regen Res. 2022;0:0.

43. Ortega SB, Torres VO, Latchney SE, Whoolery CW, Noorbhai IZ, Poinsatte K, et al. B cells migrate into remote brain areas and support neurogenesis and functional recovery after focal stroke in mice. Proc Natl Acad Sci USA. 2020;117:4983–93.

44. Doyle KP, Quach LN, Solé M, Axtell RC, Nguyen T-VV, Soler-Llavina GJ, et al. B-Lymphocyte-Mediated Delayed Cognitive Impairment following Stroke. J Neurosci. 2015;35:2133–45.

45. Shi E, Shi K, Qiu S, Sheth KN, Lawton MT, Ducruet AF. Chronic inflammation, cognitive impairment, and distal brain region alteration following intracerebral hemorrhage. The FASEB Journal. 2019;33:9616–26.

46. Costa A, Haage V, Yang S, Wegner S, Ersoy B, Ugursu B, et al. Deletion of muscarinic acetylcholine receptor 3 in microglia impacts brain ischemic injury. Brain, Behavior, and Immunity. 2021;91:89–104.

47. Jia J, Zheng L, Ye L, Chen J, Shu S, Xu S, et al. CD11c+ microglia promote white matter repair after ischemic stroke. Cell Death Dis. 2023;14:156.

48. Islam R, Vrionis F, Hanafy KA. Microglial TLR4 is Critical for Neuronal Injury and Cognitive Dysfunction in Subarachnoid Hemorrhage. Neurocrit Care. 2022;37:761–9.

49. Ge R, Tornero D, Hirota M, Monni E, Laterza C, Lindvall O, et al. Choroid plexus-cerebrospinal fluid route for monocyte-derived macrophages after stroke. J Neuroinflammation. 2017;14:153.

50. Wang R, Pu H, Ye Q, Jiang M, Chen J, Zhao J, et al. Transforming Growth Factor Beta-Activated Kinase 1–Dependent Microglial and Macrophage Responses Aggravate Long-Term Outcomes After Ischemic Stroke. Stroke. 2020;51:975–85.

51. Xu P, Hong Y, Xie Y, Yuan K, Li J, Sun R, et al. TREM-1 Exacerbates Neuroinflammatory Injury via NLRP3 Inflammasome-Mediated Pyroptosis in Experimental Subarachnoid Hemorrhage. Transl Stroke Res. 2021;12:643–59.

52. Rynkowski MA, Kim GH, Garrett MC, Zacharia BE, Otten ML, Sosunov SA, et al. C3a Receptor Antagonist Attenuates Brain Injury after Intracerebral Hemorrhage. J Cereb Blood Flow Metab. 2009;29:98–107.

53. Alawieh A, Langley EF, Tomlinson S. Targeted complement inhibition salvages stressed neurons and inhibits neuroinflammation after stroke in mice. Sci Transl Med. 2018;10:441.

54. Alawieh AM, Langley EF, Feng W, Spiotta AM, Tomlinson S. Complement-Dependent Synaptic Uptake and Cognitive Decline after Stroke and Reperfusion Therapy. J Neurosci. 2020;40:4042–58.

55. Garrett MC, Otten ML, Starke RM, Komotar RJ, Magotti P, Lambris JD, et al. Synergistic neuroprotective effects of C3a and C5a receptor blockade following intracerebral hemorrhage. Brain Res. 2009;1298C:171–7.

56. Wang Y, Zhou S, Han Z, Yin D, Luo Y, Tian Y, et al. Fingolimod administration improves neurological functions of mice with subarachnoid hemorrhage. Neuroscience Letters. 2020;736:135250.

57. Rolland WB, Lekic T, Krafft PR, Hasegawa Y, Altay O, Hartman R, et al. Fingolimod reduces cerebral lymphocyte infiltration in experimental models of rodent intracerebral hemorrhage. Experimental Neurology. 2013;241:45–55.

58. Yang Z, Dong S, Zheng Q, Zhang L, Tan X, Zou J, et al. FTY720 attenuates iron deposition and glial responses in improving delayed lesion and long-term outcomes of collagenase-induced intracerebral hemorrhage. Brain Research. 2019;1718:91–102.

59. Chu L-S, Fang S-H, Zhou Y, Yin Y-J, Chen W-Y, Li J-H, et al. Minocycline inhibits 5-lipoxygenase expression and accelerates functional recovery in chronic phase of focal cerebral ischemia in rats. Life Sciences. 2010;86:170–7.

60. Li L, Xing X, Li Q, Zhang Q, Fu L, Liu Y. Minocycline improves learning and memory functions in ischemic stroke rats via reduction of cerebral ischemia- induced neuroinflammation and apoptosis. Tropical Journal of Pharmaceutical Research. 2021;20:287–92.

61. Miao H, Li R, Han C, Lu X, Zhang H. Minocycline promotes posthemorrhagic neurogenesis via M2 microglia polarization via upregulation of the TrkB/BDNF pathway in rats. Journal of Neurophysiology. 2018;120:1307–17.

62. Hosseini SM, Gholami Pourbadie H, Sayyah M, Zibaii MI, Naderi N. Neuroprotective effect of monophosphoryl lipid A, a detoxified lipid A derivative, in photothrombotic model of unilateral selective hippocampal ischemia in rat. Behavioural Brain Research. 2018;347:26–36.

63. Xu J, Chen Z, Yu F, Liu H, Ma C, Xie D, et al. IL-4/STAT6 signaling facilitates innate hematoma resolution and neurological recovery after hemorrhagic stroke in mice. Proc Natl Acad Sci USA. 2020;117:32679–90.

64. Liu X, Liu J, Zhao S, Zhang H, Cai W, Cai M, et al. Interleukin-4 Is Essential for Microglia/Macrophage M2 Polarization and Long-Term Recovery After Cerebral Ischemia. Stroke. 2016;47:498–504.

65. Zhang Q, Zhu W, Xu F, Dai X, Shi L, Cai W, et al. The interleukin-4/PPARγ signaling axis promotes oligodendrocyte differentiation and remyelination after brain injury. Daneman R, editor. PLoS Biol. 2019;17:e3000330.

66. Wu G, McBride DW, Zhang JH. Axl activation attenuates neuroinflammation by inhibiting the TLR/TRAF/NF-κB pathway after MCAO in rats. Neurobiol Dis. 2018;110:59–67.

67. Ma L, Shen X, Gao Y, Wu Q, Ji M, Luo C, et al. Blocking B7-1/CD28 Pathway Diminished Long-Range Brain Damage by Regulating the Immune and Inflammatory Responses in a Mouse Model of Intracerebral Hemorrhage. Neurochem Res. 2016;41:1673–83.

68. Jianrong S, Yanjun Z, Chen Y, Jianwen X. DUSP14 rescues cerebral ischemia/reperfusion (IR) injury by reducing inflammation and apoptosis via the activation of Nrf-2. Biochemical and Biophysical Research Communications. 2019;509:713–21.

69. Wei J, Sun C, Liu C, Zhang Q. Effects of Rat Anti-mouse Interleukin-6 Receptor Antibody on the Recovery of Cognitive Function in Stroke Mice. Cell Mol Neurobiol. 2018;38:507–15.

70. Xie D, Liu H, Xu F, Su W, Ye Q, Yu F, et al. IL33 (Interleukin 33)/ST2 (Interleukin 1 Receptor-Like 1) Axis Drives Protective Microglial Responses and Promotes White Matter Integrity After Stroke. Stroke. 2021;52:2150–61.

71. Pettigrew LC, Kryscio RJ, Norris CM. The TNFα-Transgenic Rat: Hippocampal Synaptic Integrity, Cognition, Function, and Post-Ischemic Cell Loss. PLOS ONE. 2016;11:e0154721.

72. Xiong Xiao-Yi, Liu Liang, Wang Fa-Xiang, Yang Yuan-Rui, Hao Jun-Wei, Wang Peng-Fei, et al. Toll-Like Receptor 4/MyD88–Mediated Signaling of Hepcidin Expression Causing Brain Iron Accumulation, Oxidative Injury, and Cognitive Impairment After Intracerebral Hemorrhage. Circulation. 2016;134:1025–38.

73. Stagliano NE, Dietrich WD, Prado R, Green EJ, Busto R. The role of nitric oxide in the pathophysiology of thromboembolic stroke in the rat. Brain Research. 1997;759:32–40.

74. Chen D, Li J, Huang Y, Wei P, Miao W, Yang Y, et al. Interleukin 13 promotes long-term recovery after ischemic stroke by inhibiting the activation of STAT3. J Neuroinflammation. 2022;19:112.

75. Li J, Xu P, Hong Y, Xie Y, Peng M, Sun R, et al. Lipocalin-2-mediated astrocyte pyroptosis promotes neuroinflammatory injury via NLRP3 inflammasome activation in cerebral ischemia/reperfusion injury. J Neuroinflammation. 2023;20:148.
